# Analysis of Large Data Sets in a Physical Chemistry Laboratory NMR Experiment Using Python

**DOI:** 10.1021/acs.jchemed.3c00586

**Published:** 2023-09-19

**Authors:** Zefan Zhang, Anshul Gautam, Soon-Mi Lim, Christian Hilty

**Affiliations:** Department of Chemistry, Texas A&M University, 3255 TAMU, College Station, Texas 77843, United States

**Keywords:** Upper-Division Undergraduate, Physical Chemistry, Inquiry-Based/Discovery Learning, Computer-Based Learning, NMR Spectroscopy, Python, Data Processing

## Abstract

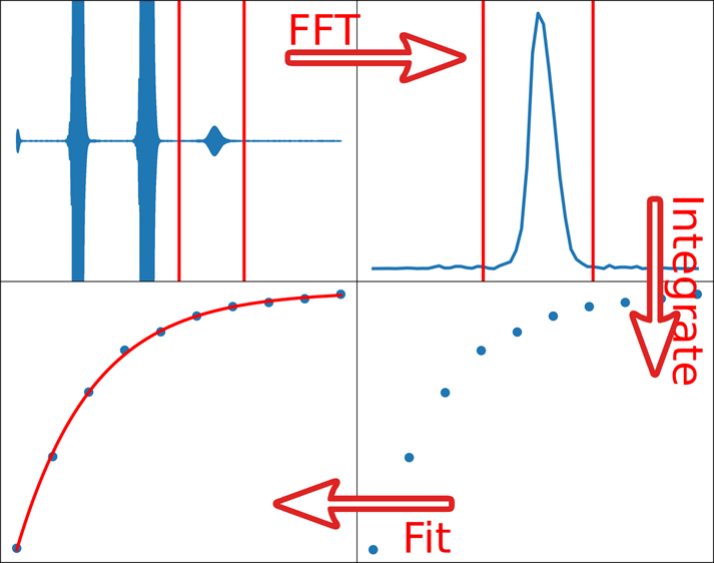

We describe an update to an experiment demonstrating low-field NMR spectroscopy in the undergraduate physical chemistry laboratory. A Python-based data processing and analysis protocol is developed for this experiment. The Python language is used in fillable worksheets in the notebook software JupyterLab, providing an interactive means for students to work with the measured data step by step. The protocol teaches methods for the analysis of large data sets in science or engineering, a topic that is absent from traditional chemistry curricula. Python is among the most widely used modern tools for data analysis. In addition, its open-source nature reduces the barriers for adoption in an educational laboratory.

## Introduction

Upper-level laboratory courses in physical and analytical chemistry are the primary venues where students in chemistry programs come in contact with measurement techniques, resulting in large data sets. These laboratories present the most natural opportunity in the curriculum to introduce modern data analysis concepts. While many data analysis and plotting tasks may be performed using familiar spreadsheet programs, these programs rapidly reach their limits in usability when handling large data sets from computer-based data acquisition.

In this communication, we describe the implementation of Python-based data acquisition and analysis for an NMR experiment in the physical chemistry laboratory. Python^1^ has risen to become one of the most popular general-purpose coding languages over the past few years. With many relevant packages, it is presently a prime choice for analysis tasks in data science.^2,3^ We further use JupyterLab,^4^ an open-source notebook software, for the interactive analysis.

Python lends itself to innovations in teaching mathematical concepts for chemistry education,^5^ creating interactive simulations,^6^ and designing new laboratories.^7^ It has been used for simulating NMR experiments to teach NMR theory with interactive graphics.^8^ In the experiment described here, implementation of the data processing tasks in Python adds a new educational element to the lab. The experiment, which we previously described in this *Journal*, has as its primary goal the introduction of the concepts of nuclear magnetic resonance.^9^ It comprises the measurement of nuclear spin precession in Earth’s magnetic field,^10,11^ the measurement of spin–lattice and spin–spin relaxation, elucidation of the origin of relaxation in stochastic processes in the sample, the effect of paramagnetic relaxation agents, and the use of pulsed field gradients for the measurement of self-diffusion. The experiment is based on a dedicated spectrometer built with a general-purpose data acquisition board. As such, it is well-suited for demonstrating basic scientific data acquisition and processing tasks using open-source Python software in addition to the goals of teaching the physical principles underlying NMR spectroscopy. This communication describes a new approach to analyze the NMR data from the experiment while exposing modern data analysis techniques that ultimately are applicable to many large scientific data sets. Working closely with the raw data, participants gain experience beyond the dedicated software that is commonly used in laboratory experiments and often tied to specific equipment. Additionally, Python and JupyterLab are free of cost for both participants and the school, thus improving access.

## Results

The laboratory experiment is organized in three parts, each performed in one weekly session. The experiment includes a data acquisition program with a graphical user interface, which is applicable to a broader range of low-field NMR experiments, including in research, and which we describe in detail elsewhere (manuscript in preparation). Some of the library functions from this program are provided to students for use with data processing, along with a set of Jupyter notebooks organized as worksheets. The notebooks (reproduced in the Supporting Information) contain partially filled sequences of code for analyzing the experimental data from each of three weekly laboratory periods. The code for the notebooks and associated Python functions are available on GitHub.^12^

**Figure 1:**
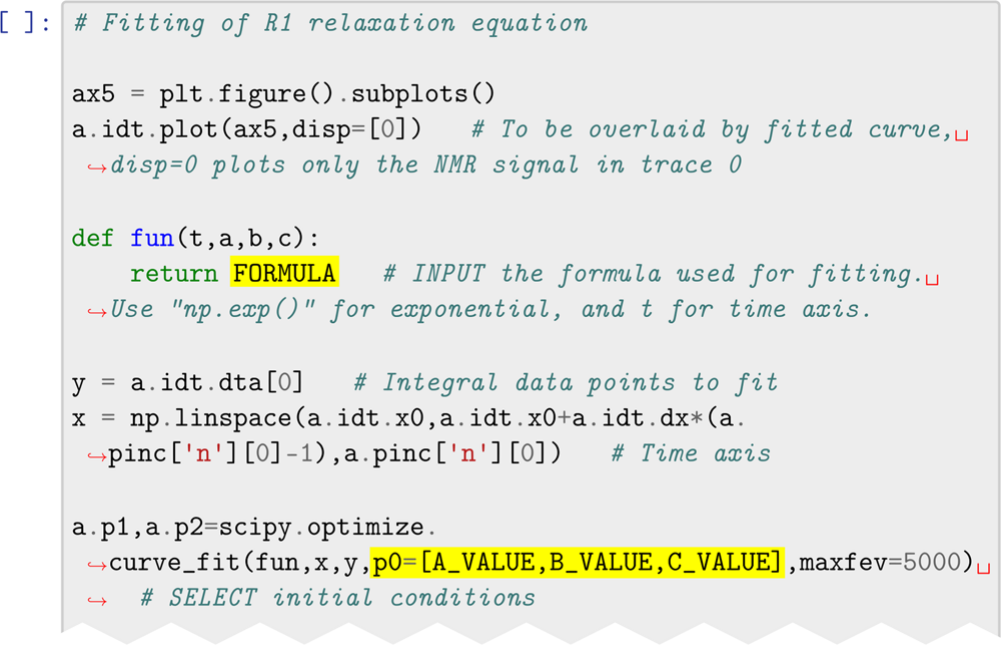
Excerpt of the fillable code provided for processing the low-field NMR data. Locations where the code needs to be completed are highlighted in yellow. The two pieces of code to be added are “a*np.exp(-b*t)+c” and “*p0 = [y[0],0.4,0]*”. The formula is rendered into Python script from NMR theory in the student’s manual. The fitting initial conditions are the expected values, which should converge rapidly.

Example code provided for part of the analysis of spin–lattice (*T*_1_) relaxation data is shown in Figure 1. This script accesses certain library functions to load data, perform Fourier transforms, and perform other experiment specific operations. Commonly used Python functions are included to produce graphs and perform calculations. A majority of the code pertaining to the data processing operations is already provided. Students are asked to fill in specific items such as formulas for calculations based on theory learned, instructions to produce well-formed graphics, and similar tasks. In principle, the amount of information provided in these worksheets can be adjusted to reflect anticipated prior knowledge in coding. We chose to provide most of the data processing functions while requiring working with the data to find information such as data ranges for processing and plotting.

**Figure 2:**
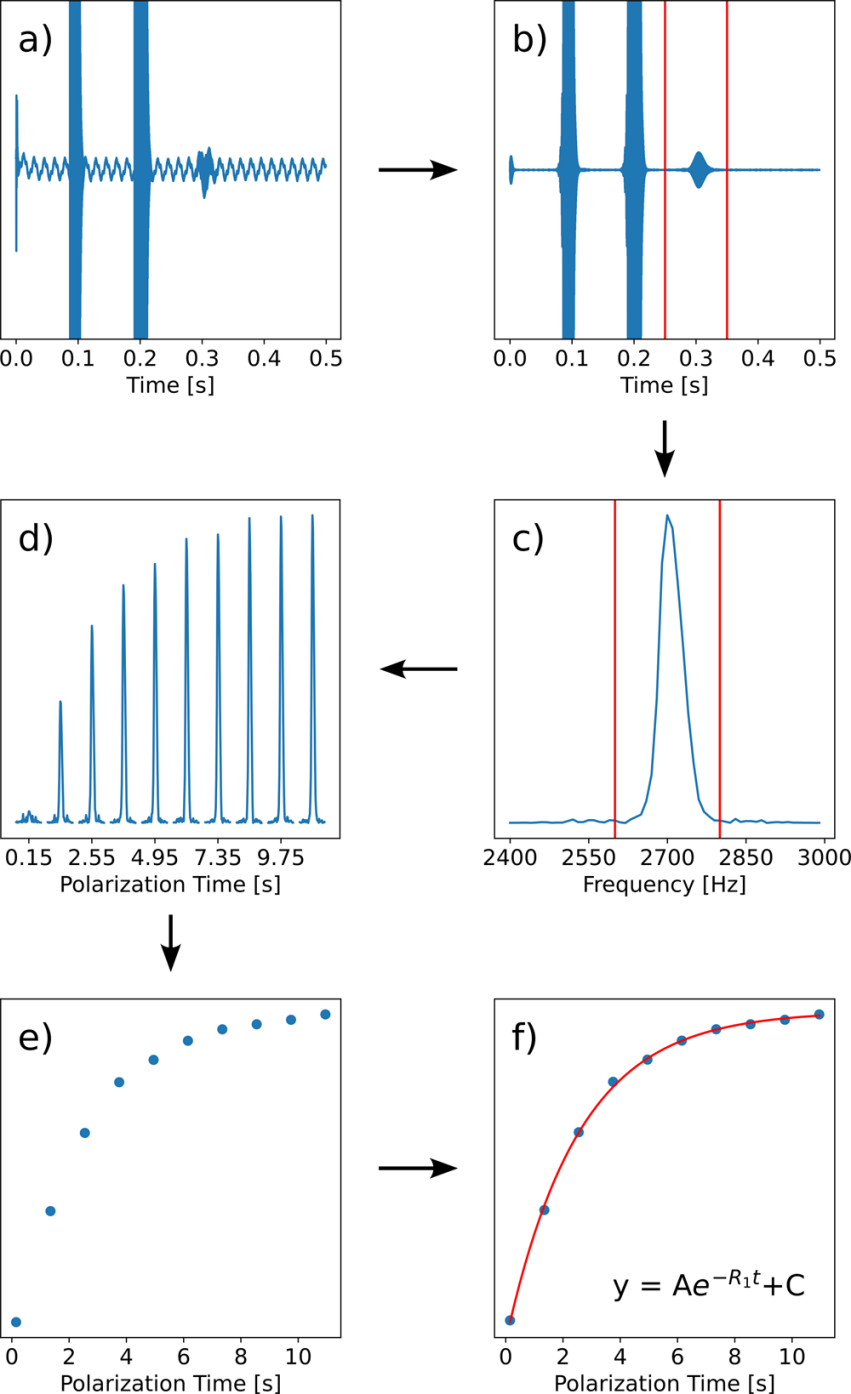
Steps and flowchart for analyzing *T*_1_ data. (a) Raw time domain data. (b) Time domain data plotted with a digital filter to visually identify the spin echo. The data range containing the echo for further analysis is indicated with vertical lines. (c) Spectrum representing the Fourier transformed echo. The range for subsequent integration is indicated. (d) Graph of a series of spectra from all measurements of the set comprising the different polarization times. (e) Integrated data points. (f) Data points fitted to the exponential function described in the theory part of the laboratory manual. The fitted result is *R*_1_ = 0.39 s^–1^.

The workflow for the entire data analysis task for *T*_1_ relaxation is shown in Figure 2. The figure illustrates the prescribed workflow for the analysis of spin–lattice relaxation (*R*_1_) data. These data are measured in the experiment by performing a series of NMR measurements comprising various lengths of time for prepolarization of nuclear spins in an elevated field produced by an electromagnet, followed by signal acquisition in Earth’s field. Without prepolarization, no detectable signal is expected. At increased polarization time, the signal is expected to rise following an exponential function, eventually reaching an equilibrium. This behavior and the underlying theory are described in the laboratory manual.

The acquired data consist of time domain signals that are sampled at a rate of several times the spin precession frequency of 2.7 kHz for a time period on the order of hundreds of milliseconds. We note that a sampling rate of 2 times the precession frequency would be sufficient to satisfy the sampling theorem; however, a higher sampling rate such as 8-fold oversampling is preferable both to reduce folded-in noise and to obtain a time-domain signal that visually appears as a smoothly oscillating waveform. As such, each signal trace consists of at least several thousand (up to ∼10,000) data points. The measured data contain signal intensity reflecting the applied radiofrequency pulses, followed by the signal from the nuclear spins in the form of a spin echo.

The first data analysis task consists of graphing the signal that is measured in the experiment (Figure 2a). Students are expected to complete instructions for producing the graph and to identify the signal portions stemming from the applied radiofrequency pulses as well as the location of the expected spin echo. Because of noise and interference signals, the spin echo may not be prominently visible in the raw data. For this reason, the analysis procedure includes the possibility of applying a time domain digital filter. The digital filter removes noise at other frequencies. The algorithm for the filter is beyond the scope of the lab; therefore, this function is already provided in the worksheet. However, students will generate the plot, which is then used to visually select the data range that includes the spin echo (Figure 2b).

After identification of the spin echo, a Fourier transform is performed to obtain a frequency domain spectrum that consists of a single peak at the spin precession frequency (Figure 2c). Again, the Fourier transform function is provided, whereas students are asked to include details about the range to be processed. This processing step is applied to every data trace in the experiment, producing a series of spectra containing a peak with an increasing intensity. A graph containing a composite image of the series of these signals, selected with the correct frequency range, is produced (Figure 2d).

Students are subsequently asked to choose suitable integration ranges and to use a provided function for integrating each of these signals (Figure 2e). The integrals plotted as a function of polarization time are expected to follow the exponential function that is described in the theory part of the laboratory manual. Because this function is related to one of the learning objectives of the lab, the function for fitting the data is not provided in the Jupyter notebook. Students are expected to write the function with the appropriate variables, allowing the use of a nonlinear curve fitting function in Python to produce a fit (Figure 2f). The *R*_1_ relaxation rate from this fit represents the result of the analysis.

The experiment includes the measurement of this relaxation rate from NMR data acquired by using multiple different samples. The obtained relaxation rates are then compared and related to the physical properties of the samples, specifically viscosity and the presence or absence of paramagnetic relaxation agents. The final analysis explains the obtained values using the theory of spin relaxation, which arises through dipolar interactions with other nuclear or electron spins and is governed through stochastic motion in liquids.^13^

An illustration of a corresponding procedure for analyzing spin–spin relaxation (*T*_2_) data is included in the Supporting Information. Additionally, worksheets for graphing data in the first week of the experiment as well as for analyzing diffusion measurements in the third week are included.

Altogether, the number of data points and number of data sets are too large to be effectively analyzed using a spreadsheet program. The experiment as described previously used a custom-made program for this purpose, which presented students with the ability to obtain the required results but did not illustrate the method for data processing step by step. The approach using Python in combination with notebooks in JupyterLab provides a means for students to perform the entire workflow for data analysis. It does not require the level of coding knowledge that would be needed for analyzing the data from first principles, which would be beyond the scope of the laboratory experiment. It also allows for including a sufficient amount of predefined code so that all tasks can be completed in a reasonable amount of time while still following each step.

The laboratory manual contains postlab questions of progressive difficulty for each day of experiments. While some of these questions address theory, others outline the steps to analyze the data with detailed information included in the Jupyter notebooks. The answers to these questions then form a basis for students to produce a lab report, which in our implementation of the class is in the format of a scientific paper.

We conducted a study to assess performance in the physical chemistry lab during the semester that coincided with the introduction of Python-based data analysis for the NMR experiment. All recruitment and study procedures were approved by the university’s Institutional Review Board (IRB). Specifically for this experiment, we assessed learning outcomes by categorizing the questions from the laboratory manual into three groups. The first group comprised the prelab questions, the second group the postlab data analysis-related questions, which require the use of Python for data processing or plotting (week 1, questions 1, 2, 4, and 5; week 2, questions 1 and 2; week 3, questions 1 and 2), and the third group the postlab questions that require interpretation (all remaining postlab questions not in the second group). The score statistics include data from 28 agreed participants in the study out of a total of 38 enrolled students. The prelab questions (group 1) had a score distribution of 91.1% ± 16.6%, the postlab data analysis questions (group 2) a distribution of 83.0% ± 17.5%, and the postlab interpretation questions a distribution of 68.9% ± 19.0%. The high scores for the prelab questions can be explained with the fact that the answers generally are found in the lab manual itself and thus appear to indicate that students were reading the lab manual prior to coming to the class. The scores for the postlab data analysis questions were somewhat lower, which indicated that the data analysis, which was explained in the lab but largely performed outside, was not trivial and required work. Nevertheless, the score indicates that students overall were successful in analyzing the data. The average for the postlab interpretation questions was the lowest of the three groups. The higher difficulty of the questions in this group can be understood on the basis that they required students to individually research and assemble information.

We found that many but not all students enrolled in the lab had previous experience with Python. For example, in our school, mathematics classes may use Python for solving equations and plotting. However, chemistry curricula do not typically include course material on computer-based processing and analysis of large data sets. This is despite the fact that these tasks have become increasingly prevalent with a reliance on data in science and engineering. The inclusion of Python-based data processing in the physical chemistry laboratory fills the need for learning relevant analysis techniques. The different levels of preparation pose some challenges in including this topic in the curriculum because every student should be able to complete the lab and have a positive learning experience. In the described implementation, this challenge is addressed by providing the annotated and partially completed worksheets. Because Python is a general purpose coding language, its use across multiple experiments or courses would be possible to further reinforce learning outcomes. Presently, in our curriculum, Python is used in another experiment in computational chemistry in the second semester of the physical chemistry lab.

A technical challenge in the use of Python for data processing is that there are multiple ways to install and use the software. Different Python distributions exist, which include among others the official installer downloaded from the Python website, installers in operating systems’ app stores or repositories, and others. At our school, Python and Jupyter are further available preinstalled in the student computer laboratories. For this experiment, students were allowed to choose their preferred method of installing or accessing the software (see the laboratory manual). Students liked to use their preferred method if they had previous experience with Python. However, multiple choices can cause a heterogeneous set of issues, including how to copy data on different systems, how to install packages, and how to run the Python interpreter. In our lab, students worked in small groups of a maximum of four participants supervised by a teaching assistant. In this format, it was possible to provide individual assistance to deal with such issues. In other uses with larger groups, we suggest prescribing a single method for accessing the software, which would make it easier to anticipate the technical issues that students will need help with.

Apart from the above-mentioned significant educational benefits of learning techniques for the analysis of large data sets, the use of Python in a physical chemistry laboratory presents several advantages. First, all of the external software used in this experiment is freely available and open-source. This not only eliminates licensing fees for operating the laboratory but also ensures that there are no financial barriers for every student to be able to install the software on their own computer. Second, the compatibility of JupyterLab with Python allows for an easy way to provide an interactive notebook for students to work with. Third, the current popularity of this software results in a large online user community willing to provide assistance as well as high-quality online documentation.

## Hazards and Safety Precautions

The data acquisition and processing methods described do not introduce additional hazards apart from those intrinsic to the experiment. Operating the low-field NMR spectrometer presents electrical hazards. The spectrometer should not be operated when wet, and metal leads or metal parts should not be touched during operation. Chemicals used in the experiments, including organic solvents and free radicals, are harmful. Personal protective equipment must be worn at all times when handling the chemicals.

## Conclusions

In summary, we have developed a Python-based module for a data-intensive NMR experiment in the physical chemistry laboratory. The laboratory experiment provides a means of teaching data analysis with this widely used software tool, a topic that is not sufficiently presented in chemistry curricula. Notebooks organized as worksheets are provided that assist students with differing prior skills to complete the tasks assigned with the experiment. The open-source nature of the software reduces barriers for its use by all students on their own computer. A similar model could be adopted for various experiments in physical and analytical chemistry laboratory experiments.

## Supporting Information Available

Flowchart for analyzing *T*_2_ data, laboratory manual, and Jupyter notebooks provided to students (PDF)
