# Supplementary material for: Analysis of Large Data Sets in a Physical Chemistry Laboratory NMR Experiment Using Python
Source: J Chem Educ. 2023 Sep 19;100(10):4109–13. doi: 10.1021/acs.jchemed.3c00586 (PMC10862468; doi:10.1021/acs.jchemed.3c00586)
Supplement: Supplementary file 1 — ed3c00586_si_001.pdf [file ed3c00586_si_001.pdf]

Supporting Information for

Analysis of Large Data Sets in a Physical Chemistry Laboratory NMR Experiment using Python

Zefan Zhang, Anshul Gautam, Soon-Mi Lim and Christian Hilty*

Chemistry Department, Texas A&M University, 3255 TAMU, College Station, TX 77843

*email: chilty@tamu.edu

Table of Contents

Steps and flow chart for analyzing T_2 data........2
Low-Field NMR Laboratory Manual (‡)........3
Notebook for week 1........33
Notebook for week 2. T_1 relaxation experiment........37
Notebook for week 2. T_2 relaxation experiment........41
Notebook for week 3. Diffusion experiment........45

(‡) The updated laboratory manual provided to students includes the instructions for data analysis using Python (references reformatted to ACS format). Original version is in: Hilty, C.; Bowen, S. An NMR Experiment Based on Off-the-Shelf Digital Data-Acquisition Equipment. *J. Chem. Educ.* **2010**, *87* (7), 747–749.

Steps and flow chart for analyzing T_2 data

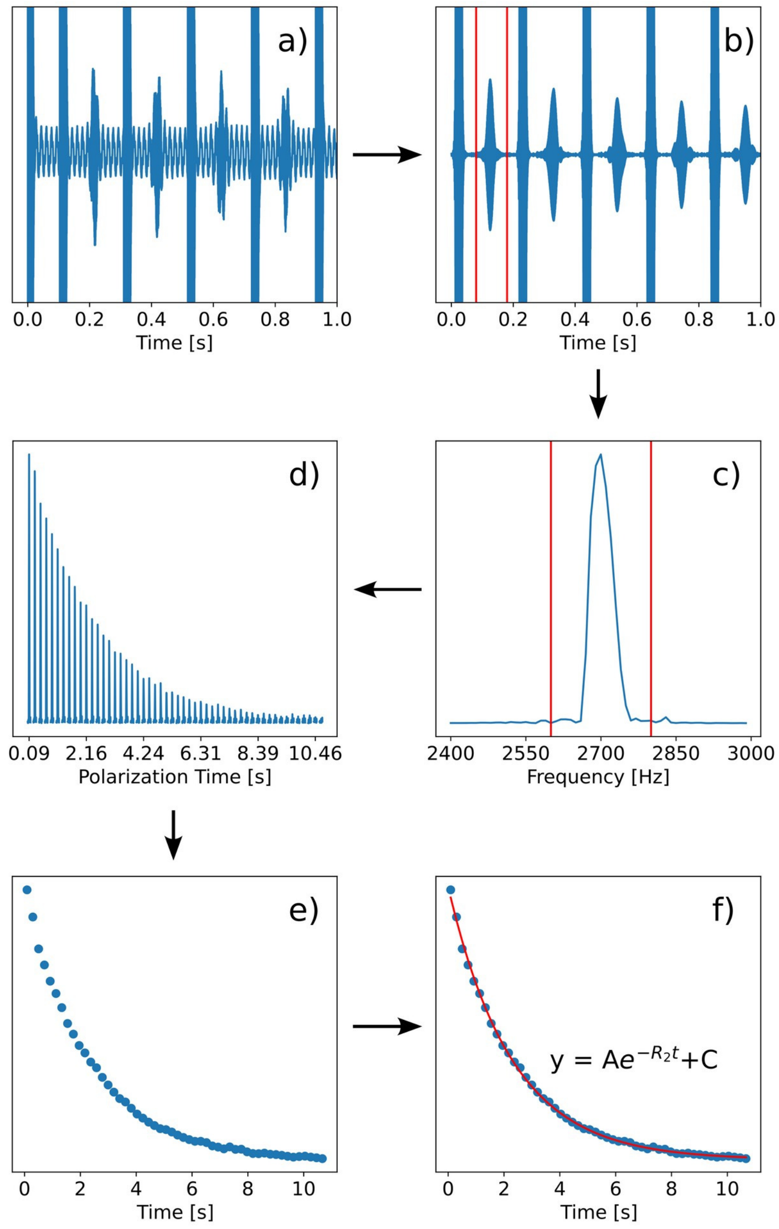

Figure S1. Steps and flow chart for analyzing T_2 data. a) Raw time domain data. b) Time domain data plotted with digital filter. The data is split into multiple slices for each loop in the spin-echo (CPMG) pulse sequence, with the first excitation pulse and waiting time curtailed. In the first slice, the data range containing the echo for further analysis is indicated with vertical lines. c) Spectrum representing Fourier transformed echo. The range for subsequent integration is indicated. d) Graph of a series of spectra from all slices comprising the progressing time axis. e) Data points from integrated signals. f) Data points fitted to the exponential function described in the theory part of the laboratory manual. The fitted result is R_2 = 0.44 s^{-1}.

Low-Field NMR Laboratory Manual

Concepts: Nuclear magnetic resonance, spin, relaxation, diffusion, data acquisition

Introduction

Nuclear magnetic resonance is a versatile technique that can be used both for acquiring chemical shift resolved spectra to determine molecular structure, and to measure the spatial distribution of spin density, to obtain the magnetic resonance image (MRI) of an object. In both cases, the precession frequencies of nuclear spins are measured. In spectroscopy individual sites in a molecule have different frequencies because of microscopic variations in the magnetic field brought about by the molecule itself. In MRI, a spatially dependent magnetic field ("field gradient") is applied externally, in order to determine the spatial position of spins based on their frequencies.

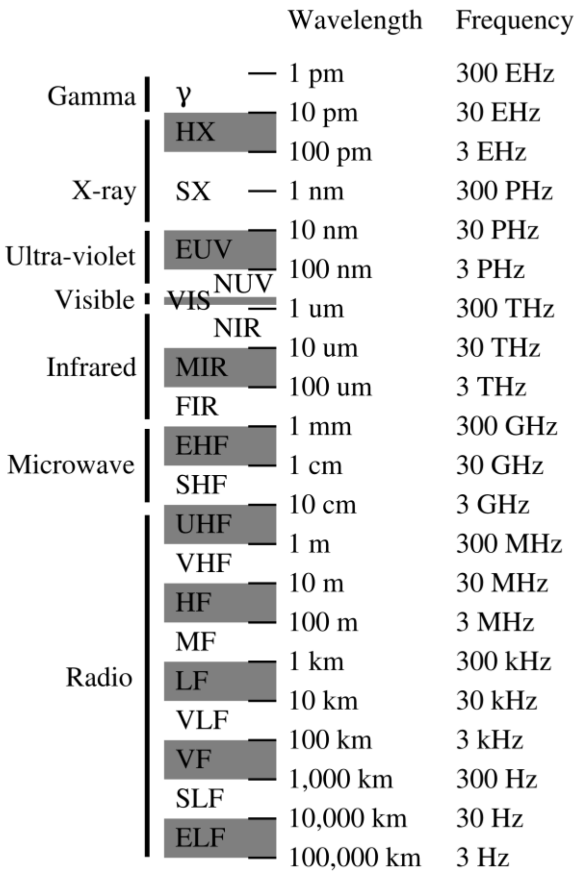

Figure 1. Electromagnetic Spectrum. The visual range is approx. 400 nm (violet) to 700 nm (red). Typical high-field NMR experiments are performed in the UHF band. The earth-field NMR in this experiment takes place in the VF band.

The purpose of this experiment is to illustrate the underlying principles of NMR. The experiments are performed on an NMR spectrometer operating at low frequency (Figure 1) in the earth's ambient magnetic field. They give a hands-on introduction into pulsed NMR instrumentation, using a spectrometer representing all of the features of a modern NMR spectrometer in a simplified manner, without the need for complex radio-frequency electronics. Finally, they also introduce data acquisition using python and jupyter.

Theory

The Zeeman Effect

Magnetic resonance is based on the observation that particles with a spin s carry an associated magnetic moment

$$\vec{\mu} = \gamma \vec{s} \, , \tag{1}$$

where γ is called the gyromagnetic ratio (1-4). The gyromagnetic ratio is specific to each nucleus (see the Constants section). The Zeeman effect describes the energy of such a particle when it interacts with an externally applied magnetic field (Figure 2):

$$E = -\vec{\mu} \cdot \vec{B} \, . \tag{2}$$

In order to evaluate the scalar product in equation 2, we need to multiply μ_z, the projection of μ on the magnetic field vector, with B. Because

$$s_z = \hbar m_z \, , \tag{3}$$

we obtain

$$\mu_z = \gamma \hbar m_z \, , \tag{4}$$

and

$$E = \gamma B \hbar m_z \, . \tag{5}$$

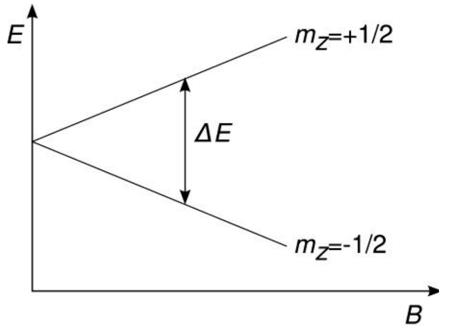

Figure 2. Zeeman effect for spin-1/2 system.

For a particle with s = 1/2, such as a proton, m_z can only take the two distinct values +1/2 or -1/2, describing the two spin-states that are often called the “spin-up” and the “spin-down” state. s and m_z describe the state of the particle completely; due to the uncertainty relation, we cannot know, for example, the projection of s onto a different axis (e.g. m_x) at the same time.

In order to change between the two spin states, m_z needs to change by one unit. The frequency f (and the corresponding angular velocity ω) of such a transition is given by the relation

$$\Delta E = \hbar \omega = hf \, . \tag{6}$$

In a semi-classical picture, the spin is said to precess on the surface of a cone around the magnetic field vector (Figure 3). By combining equations 5 and 6, we can see that the precession frequency is ultimately given by the magnitude of the magnetic field and by the gyromagnetic ratio.

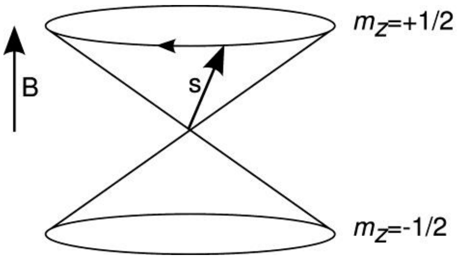

Figure 3. Spin precession.

Polarization

A spin in a magnetic field preferentially occupies the lower of the Zeeman levels, in equilibrium with the temperature (Boltzmann distribution). The spin system is thus said to be polarized, with polarization defined as

$$p = \frac{n_{+1/2} - n_{-1/2}}{n_{+1/2} + n_{-1/2}}, \quad (7)$$

where $n_{+1/2}$ and $n_{-1/2}$ represent the number of spins on the respective levels.

At equilibrium,

$$p_{eq} = \frac{1 - e^{-\Delta E/(kT)}}{1 + e^{-\Delta E/(kT)}} = \tanh\left(\frac{\Delta E}{2kT}\right). \quad (8)$$

The spin system attains its equilibrium polarization over a certain amount of time after it is brought into a magnetic field. This process is mediated by energy exchange with molecular degrees of freedom that are external to the spin system, such as rotation and vibration. It is called "spin-lattice" relaxation (the "lattice" is the ensemble of motional degrees of freedom of the molecule). The spin polarization builds up asymptotically to the equilibrium value, with a time constant T_1:

$$p = p_{eq}\left(1 - e^{-t/T_1}\right) \quad (9)$$

An NMR experiment always measures a signal corresponding to the sum of all spins, *i.e.* proportional to the total magnetization vector

$$\vec{M} = \sum_i \vec{\mu}_i\ . \quad (10)$$

The first two columns in Figure 4 illustrate the transition from a disordered to a polarized spin system. Immediately after the spin system is brought into the magnetic field, spins are equally distributed over the two energy levels, and they precess with random phase. This is the disordered state, where the total magnetization $M = 0$. In the polarized state, different numbers of spins occupy the two levels, but they still precess with random phase. In this case, the sample has a total magnetization aligned with the z-axis of the coordinate system. Although the equilibrium state is an ordered state, it is constant in time. It does not produce a signal in an NMR experiment, where inductive coupling of the magnetization to a receiver coil is used.

Coherence

A coherence is generated by a radio-frequency (RF) pulse that is applied to a spin system, which initially is for example in the polarized state M_z.

$$\vec{B}_{rf} = \vec{B}_1 \cos\left(\omega_{rf} t + \varphi\right) \quad (11)$$

By coupling of the spin to the electromagnetic field, a transition between energy levels can be achieved if the frequency of the pulse corresponds to the precession frequency of the spins. The spin system is then in resonance with the applied field. After such an RF pulse, all of the spins affected by the pulse precess with the same phase (illustrated in the third column of Figure 4). This is called a coherence.

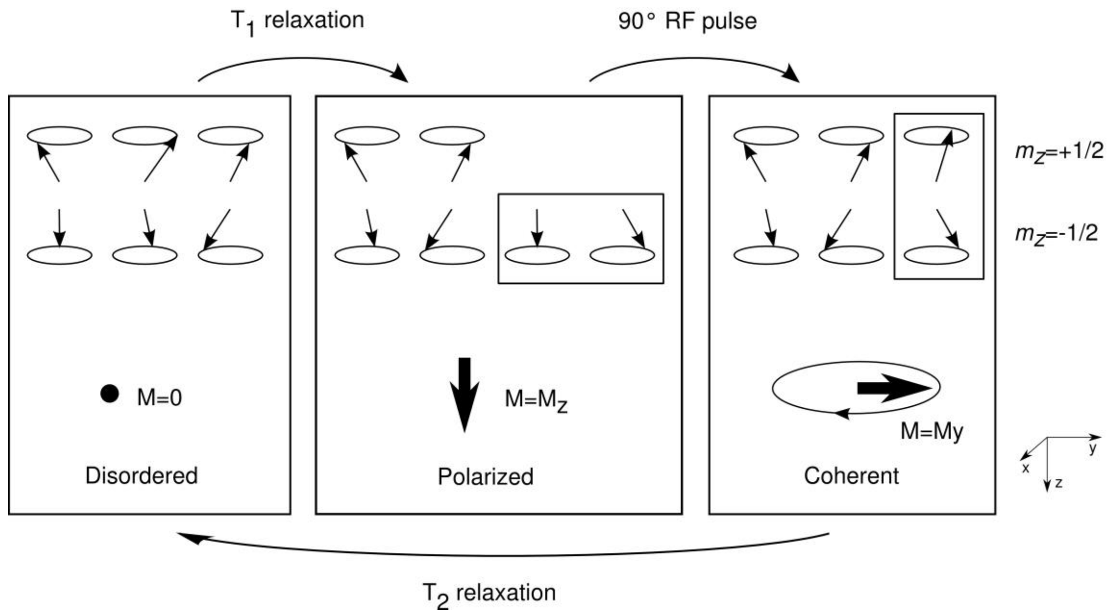

Figure 4. Coherence and magnetization. A polarized state is achieved by T_1 relaxation in a magnetic field. From the polarized state, a 90° pulse generates an NMR observable coherence. This coherence dephases due to T_2 relaxation. (Note that in the coherent state, T_1 and T_2 relaxation occur simultaneously, so the spin system bypasses a true disordered state when going from the coherent to the polarized state.)

A 90° pulse is a pulse which changes the orientation of the magnetization vector by 90°, from M_z to M_x. Once the magnetization vector is aligned along the x-axis, it precesses in the x-y plane with the same frequency as the spins. This time-varying magnetization is observable by a typical NMR experiment, where inductive coupling to a receiver coil is used to detect the signal.

A coherence decays over time due to small variations in the magnetic field, which make some spins precess slightly faster than others. The spin system then returns to the disordered state. This decay is called "spin-spin" relaxation. It contains a contribution from microscopic fluctuations within the molecule, and a contribution from macroscopic external field variations. The microscopic contribution alone is often denoted with a time constant T_2, and the sum of all contributions with T_2^*. The magnetization thus depends on an exponential function of time:

$$M = M_0 e^{-t/T_2^*} \tag{12}$$

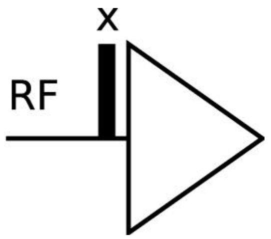

Figure 5. One-pulse experiment. The black bar represents a 90º RF pulse with phase x (φ=0 in equation 11). The triangle symbolizes data acquisition.

The macroscopic contribution is often large, and can be re-focused with a spin-echo experiment (see below).

An NMR Experiment

The steps of a complete pulsed NMR experiment are already depicted in Figure 4. Figure 5 shows the same experiment in symbolic notation. Briefly, a polarized spin state is achieved by thermal equilibration of the spin system in a magnetic field. Subsequently, an NMR observable coherence is generated with a 90º RF pulse. While the coherence relaxes back to the polarized state, the NMR signal is picked up in the form of a small oscillating voltage (typically in the microvolt range) that is induced in a receiver coil by the precessing magnetization vector *M*. The signal is called a free induction decay (FID). The attainable signal after generation of a coherence is directly proportional to the level of polarization.

In a high-field NMR experiment, differences in precession frequency (chemical shift) for different spins in a molecule are measured, yielding the NMR spectrum of a substance. Because the difference in precession frequency is proportional to the field strength, the low field NMR experiments that you will carry out here are not able to distinguish between chemical shifts. They are measuring other parameters describing the sample, such as T_1 and T_2 relaxation, diffusion or spatial distribution of spin density.

Time vs. Frequency domain

An FID is acquired as intensity as a function of time. The signal generated from one type of spins is a sinusoidal oscillation. Since each type of spins is associated with a particular resonance frequency, a more suitable form of NMR data presentation is in the form of a spectrum. A spectrum presents intensity as a function of frequency.

The mathematical operation that converts data from time domain to frequency domain is a Fourier transform.

$$g_k = \sum_{n=0}^{N-1} f_n e^{-\frac{2\pi i k n}{N}}. \tag{13}$$

f_n is the time-domain data series, g_k the frequency domain series, N is the total number of points, and n and k are indices. Because the signal is only measured at discrete points in time, equation 13 shows the discrete Fourier transform, which entails summation. An alternate form of the Fourier transform for a continuous mathematical function would use an integral instead. The Fourier transform is reversible. Its inverse is:

$$f_n = \frac{1}{N}\sum_{k=0}^{N-1} g_k e^{\frac{2\pi i k n}{N}}. \tag{14}$$

Therefore, the time-domain representation (FID) and the frequency-domain representation (spectrum) of the NMR data are completely equivalent.

An important theorem by Nyquist states that for a given sampling interval t_{dw} (= dwell time; time between two acquired data points), the maximum frequency ("Nyquist frequency") that can be detected is

$$f = \frac{1}{2t_{dw}} \tag{15}$$

Knowing the frequency of nuclear spins to be detected, this relation gives the maximum allowable sampling interval. Intensity from signals that have a higher frequency than the

Nyquist frequency is however not lost. Rather, it is aliased back into the spectrum and appears at a different frequency that is lower than the Nyquist frequency. The NMR experiment uses filters to prevent higher-frequency noise from being aliased into the spectrum.

Note that in the low-field experiments, we typically observe a single peak. However, it is still advantageous to use a Fourier transform for data analysis, because it is easier to measure the signal intensity from a peak in the spectrum rather than from the oscillation in the FID.

Molecular Motion and Spin-lattice Relaxation

Spin-lattice relaxation, or the return of the spin population to the equilibrium, is caused by magnetic field fluctuations at the location of a nuclear spin. These field fluctuations drive the population to equilibrium by inducing transitions between the Zeeman levels. Molecular motions (in the "lattice", hence the name) generate local field fluctuations via different mechanisms. For example, the field produced by the magnetic moment of a nearby spin fluctuates as the distance vector to that spin changes (Figure 6). If the nearby spin s_2 is another nuclear spin, this mechanism gives rise to one of the most common spin relaxation mechanisms called dipolar relaxation. If s_2 is an unpaired electron spin from a radical or from metal ion, the relaxation mechanism is called paramagnetic relaxation enhancement (PRE). The PRE effect is larger than nuclear dipolar relaxation because the electron spin has a larger magnetic moment than a nuclear spin.

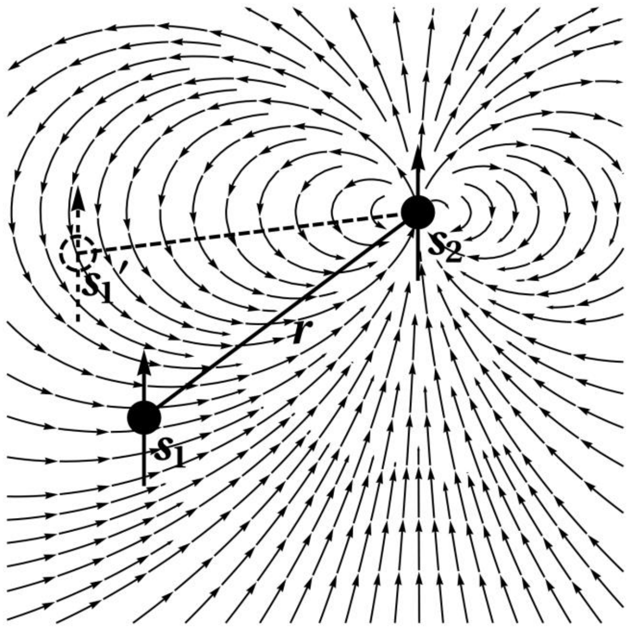

Figure 6. Local magnetic field of a spin s_2, affecting spin s_1 and causing relaxation as the orientation of the distance vector r fluctuates due to random molecular motion. Note that regardless of the molecular orientation, the dipole moments are always aligned with the external magnetic field.

In order to quantify relaxation, it is necessary to describe the field fluctuations mathematically. Molecular motions are random, meaning that the exact time dependence of the field they cause cannot be known. Fortunately, in order to quantify relaxation, it is sufficient to know on average the magnitude of fluctuations at the frequency corresponding to the Zeeman transition to be induced. The following briefly describes how this can be achieved (5, 6).

The process giving rise to relaxation can be described by a random variable *f* expressing the fluctuating part of the magnetic field. *f* is defined such that its average over time is 0. It can be characterized by its time correlation function, which quantifies the certainty with which the value of *f* at a time $t + \tau$ can be predicted, if it is known at time *t*. Mathematically, it is given as the autocorrelation of *f*:

$$G(\tau) = \int_{-\infty}^{\infty} f(t)f(t+\tau)dt \tag{16}$$

$G(\tau)$ should be largest for $\tau = 0$, and should decay to 0 as τ increases. This is because it becomes increasingly difficult to predict *f* in the more and more distant future. How rapidly $G(\tau)$ decays towards 0 depends on how rapid the fluctuations are. Based on this discussion, a plausible explicit form for the time correlation function is

$$G(\tau) = G_0 e^{-|\tau|/\tau_c} \tag{17}$$

The parameter τ_c, also called the correlation time, characterizes the random fluctuations. It can be related to the macroscopically observable viscosity η and the hydrodynamic radius r, using an equation by Debeye (for dipolar relaxation):

$$\tau_c = \frac{4\pi\eta r^3}{3kT} \tag{18}$$

Expressed in words, the larger the viscosity, or the bulkier the molecule, the slower the molecular motions become, and the longer τ_c is.

A theorem by Wiener and Khinchin states that the frequency spectrum (more precisely, the power spectral density) of the fluctuations is given by the Fourier transform of the time correlation function. Here, this is

$$J(\omega) = \mathrm{Re} \int_{-\infty}^{\infty} G(\tau)e^{-i\omega\tau}\, d\tau = 2G_0 \frac{\tau_c}{1+\omega^2\tau_c^2} \tag{19}$$

$J(\omega)$ describes how much power the fluctuating field can supply to induce a transition at the angular frequency ω, and therefore how efficiently it can cause relaxation. The relaxation rate is proportional to $J(\omega)$:

$$R_1 = \frac{1}{T_1} = 4G_0 \frac{\tau_c}{1+\omega^2\tau_c^2} \tag{20}$$

(The additional factor of 2 arises from the fact that every spin that is removed from one Zeeman level is added to the other, and can be found when explicitly writing the rate equations).

Examining R_1 on a logarithmic scale (Figure 7) shows that the spin-lattice relaxation rate is largest for small ω, and drops rapidly after a certain corner frequency has been reached. This is because the molecular motions do not create significant fluctuations above this corner frequency, which can further be seen to increase with decreasing τ_c. In the present experiments, all measurements made in the field of the earth are well below this corner frequency. In this case, the relaxation rate simply increases if molecular motions become slower (see the different curves for increasing τ_c in the figure).

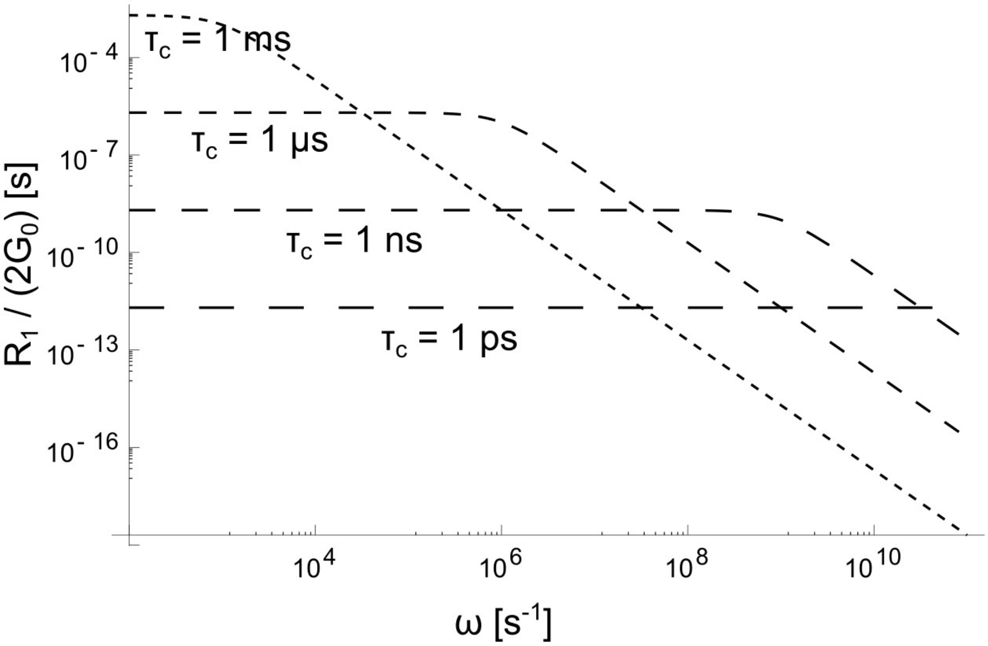

Figure 7. Double logarithmic plot of R_1 in function of ω, for different values of τ_c.

Pulsed field gradients

Some of the NMR experiments carried out here make use of pulsed field gradients (PFG). PFG's are controlled disturbances in the magnetic field. For the duration of such a "gradient pulse", the magnetic field varies linearly with position in the sample:

$$B = B_0 + zG \tag{21}$$

where B_0 is the constant magnetic field, z the position and G the gradient strength. During a field gradient, magnetization precesses with different frequency in different parts of the sample:

$$\omega = \omega_0 + \gamma zG \tag{22}$$

If the field gradient is sufficiently strong, the experiment in Figure 8 does not yield a signal because phase coherence has been lost. It is important to note that the state of the system is not truly random, as there is the known dependence of the magnetic field on the position in space, and the coherence could be refocused ("brought back") with a gradient pulse of opposite magnitude. You will use pulsed field gradients for the diffusion experiments.

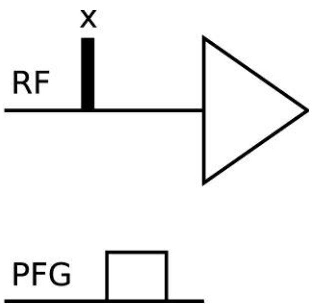

Figure 8. Experiment demonstrating the effect of a pulsed field gradient.

Major Equipment

The experiment uses a low-field NMR spectrometer, the main components of which are illustrated in Figure 9. A more detailed diagram is given in Figure 10 (7, 8). There is one RF channel, two pulsed field gradient channels and a channel for generating the polarization field (9, 10).

Radio Frequency Channel

The core of the spectrometer is a resonant circuit, composed of the NMR coil with inductance L_c and the tuning capacitor C_T. This circuit is similar to a radio antenna; it resonates at a specific frequency

$$\omega = 2\pi f = \frac{1}{\sqrt{L_c C_T}} \qquad (23)$$

by transferring electrical energy back and forth between the inductance and the capacitance. It is used both for transmitting RF pulses and for detecting the FID signal from precessing spin magnetization of the sample. The coupling between the sample magnetization and the coil is efficient only if the resonance frequency is equal to the spin precession frequency. The function of the variable tuning capacitor is to allow adjustment of the resonance frequency, according to equation 23.

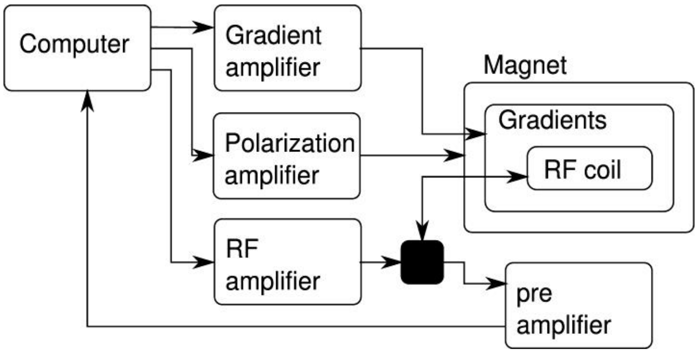

Figure 9. Schematics of the spectrometer. The arrows represent the flow of signals from the computer via the amplifiers to the coil and back. The black box is the transmit/receive switch (cross-diodes).

The RF pulse is synthesized in the computer using a digital-to-analog converter of a data acquisition board (National Instruments PCIe-6259) and amplified with the RF power amplifier (AE Techron LVC 623). The power amplifier is symbolized in Figure 10 with a triangle. Subsequently, the FID signal is amplified using the RF pre-amplifier (Stanford Research 560) and detected in the computer using an analog-to-digital converter. The pre-amplifier is capable of amplifying a voltage by a factor of 10^4, and thus allows observing a signal in the μV range. The RF power amplifier would contribute an excessive amount of noise during signal acquisition. It is thus decoupled from the resonant circuit and the pre-amplifier by the transmit-receive switch. The transmit-receive switch is realized by the crossed diodes D_1 and D_2, which conduct only during a pulse, when the RF voltage from the amplifier exceeds the 0.7 V forward voltage of the diodes. Further, the pre-amplifier is protected from voltage spikes by the Zener diodes Z_1 and Z_2.

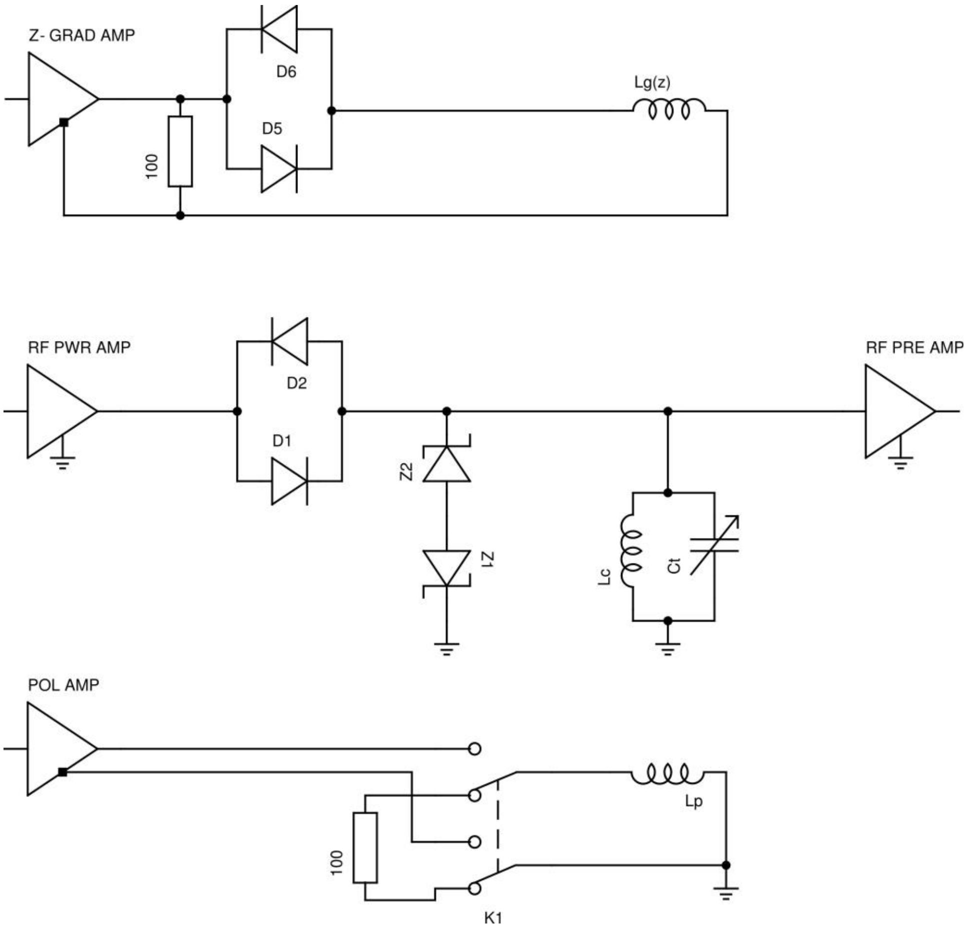

Figure 10. Circuit diagram of the spectrometer. Top to bottom: z gradient channel, radiofrequency (RF) channel, polarization channel.

Polarization Channel

The polarization channel drives the large polarization coil L_p with up to 14 A of current. During the time that the polarization field is turned on, the spin system polarizes according to equation 9. This spin-polarization will allow observing an NMR signal. The polarization field is turned off during the NMR experiment to take advantage of the superior homogeneity of the magnetic field of the earth. The polarization current follows a signal from a digital-to-analog converter on the data acquisition board, and is ramped down slowly to preserve spin magnetization (adiabatic change in field) immediately before the start of an experiment. After the polarization field is ramped down, the relay K_1 switches so that the coil is shorted over a 100 Ω resistance.

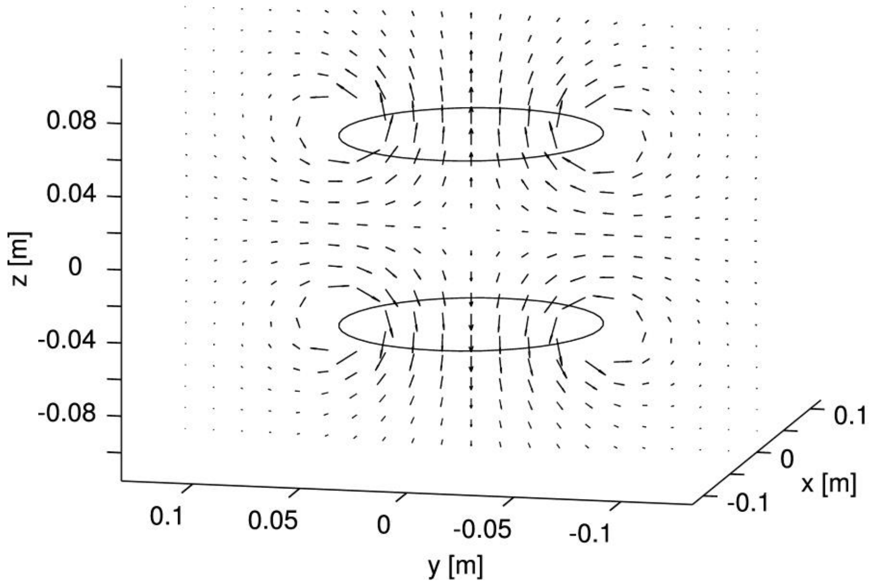

Figure 11. Maxwell pair gradient coil. Current is flowing counterclockwise in the upper loop and clockwise in the lower loop. The arrows represent the magnetic field in the plane x=0.

Pulsed Field Gradient Channel

The z-gradient channel consists of a power amplifier and a z-gradient coil. The z-gradient coil is in a "Maxwell pair" arrangement, where two parallel current loops of opposite direction form the magnetic field (Figure 11 and Figure 12) (11). The z-gradient channel also possesses a passive blanking circuit consisting of the diodes D5 and D6, and of the two 100 Ω resistors.

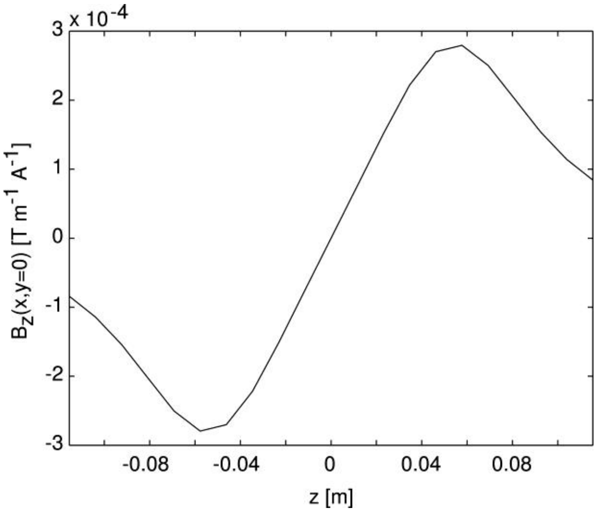

Figure 12. z-component of the gradient field from the Maxwell pair coil along the line x=0 and y=0. The coil position is ±0.05m; the usable linear region is between z=±0.04m.

Experiments

Safety

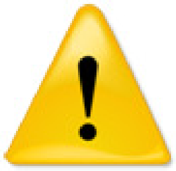

Electrical: The power amplifiers are capable of producing up to 20 A of current and in some cases voltages of at least 120 V. This presents a burn and electric shock hazard. Always turn off the amplifiers and wait 10 sec. before changing connections. Do not touch any metal parts of the equipment during operation, as they could carry electrical current. The magnet assembly must be properly grounded.

Chemicals: Toluene (CAS# 108-88-3) - Harmful or fatal if swallowed. Harmful if inhaled or absorbed through skin. Vapor harmful. Flammable liquid and vapor. May affect liver, kidneys, blood system, or central nervous system. Causes irritation to skin, eyes and respiratory tract. Glycerol (CAS# 56-81-5) - Harmful if inhaled, absorbed through the skin or swallowed. May cause irritation to skin, eyes, and respiratory tract. May affect kidneys. Gadolinium(III)-diethylenetriaminepentaacetic acid (Gd-DTPA) (CAS# 80529-93-7) - Harmful if inhaled, absorbed through the skin or swallowed. Causes skin and serious eye irritation. May cause respiratory tract irritation.

Preventing Damage

- No high-power transmitting cables (including the RF, gradient and polarization coil cables) may be directly connected to signal cables from the computer.
- The maximum allowable power for the RF amplifier is a setting of 12 (knob on channel 2 of the upper amplifier), together with an input signal of 1 V pk-pk (defined in the software).

Preparations

- For each lab class, please bring a USB memory stick to save data.
- Python (https://www.python.org) and Jupyter (https://jupyter.org) are used for data processing. You will need to install Python and Jupyter on your computer or use the version installed at the TAMU Open Access Lab. For more information, see the appendix.

Startup and Shutdown

- The red "main power" button controls the entire system. Individual power buttons are located on the following units: SR-560 pre-amplifier: on the front panel. Computer: On the front. Power amplifiers: on the front panel.
- When leaving the system, the two power amplifiers should be turned off. All of the other components may be left on.

Week 1: Instrumentation

In this first lab, you will get to know the low-field spectrometer by setting up the hardware, acquiring an FID and determining the 90º pulse length. There is also an introduction to data acquisition and analysis using Python and Jupyter.

Prelab Questions

1. Write the formula for calculating the resonance frequency from the magnetic field.
2. Calculate the resonance frequency for protons in the earth's magnetic field, as well as in a field of 10 T.

Placement of the NMR spectrometer

The axis of the RF coil needs to be oriented perpendicular to the earth's magnetic field, since the B_1-field of the RF-pulse (equation 11) needs to fulfill this condition in order to excite the spin system. To find the direction of the magnetic field, measure all three components of the ambient magnetic field using the Hall probe (F.W. Bell) and the three-axis Plexiglas block. Subsequently, place the spectrometer in the correct orientation.

Set up the hardware of the spectrometer according to Figure 10. The coil is connected to the power amplifier via cross-diodes and connected directly to the pre-amplifier. On the pre-amplifier side, the box with the capacitor also contains the cross-diodes and two Zener diodes to shunt high transient voltages.

Tuning the coil

Based on the measured ambient magnetic field, calculate the approximate resonance frequency for protons. You will need this frequency to tune the coil.

Start the daqapp2.py program (right click and select Open with > Python). In the program, select File/Load. Navigate to the data folder and load tuning.json. Run the program by clicking on the blue arrow in the toolbar. Running the program excites the coil with a small voltage, which is subsequently removed. At that time, the circuit will resonate. The resonance frequency of the circuit can thus be deduced. Set the pre-amplifier gain so the oscillation is not clipped at the upper or lower edge of the observable voltage range. After each change, re-acquire a tuning signal by clicking on the blue arrow. To see the frequency spectrum of the oscillation, click on the Display Frequency button, . Refer to Appendix 2 for display functions and parameters that can be changed.

Adjust the variable capacitor using its dip switches until the circuit resonates at the calculated precession frequency of the nuclear spins. Save the signal using File/Save.

Determining the length of a 90º pulse

The simplest NMR experiment is given in Figure 5, where a 90º pulse converts longitudinal magnetization (M_z) into detectable transverse magnetization (M_x). That experiment is however not suitable for the low-field spectrometer because the typical magnetic field inhomogeneities in the laboratory shorten the apparent T_2 (a.k.a. T_2*) to below the coil ringdown time. The coil ringdown time is the time it takes the resonant circuit to recover from an RF pulse, *i.e.* the length of time that the tuning signal lasted in the previous section. Therefore, a spin-echo experiment is used. In a spin-echo experiment, a 180º pulse follows

the 90º pulse (Figure 13). The spin coherence is thus refocused after a delay of 2Δ. However, in order to obtain signal using this experiment, you need to determine the length/power relationship of the 90º pulse.

Set the parameters:

- On the pre-amplifier, select DC coupling, "Low Noise" gain mode, gain of 10^3-10^4, and high and lowpass filters that most closely match the resonance frequency.
- On the RF amplifier, select a power level (below 12 on the dial).
- Review the steps in Appendix 2 to run an experiment using the data acquisition program.
- Load an experiment for measuring a spin echo using the pulse program named cpmg. Enter the resonance frequency determined in the previous section, and adjust other parameters as needed (Appendix 2). Use at least 8x oversampling (*i.e.,* 16 samples per period).
- Review the pulse program display to make sure the pulse program looks correct.
- Place a sample (ca. 400 ml water) in the coil and run the experiment once. During the experiment, you should see some flashing lights on the amplifiers and the pre-amplifier, and you should hear a clicking noise originating from the relay in the pre-polarization circuit.
- Check whether a signal is obtained. When looking at an FID, be sure to identify the spin-echo (rising and falling signal) and distinguish it from the larger signal generated by the RF pulse and coil ring down. Compare to the display in Figure 14.
- The Iteration parameters allow you to automatically repeat the experiment while iterating a parameter. In the Iteration params window, set p1 to be iterated, and select an increment value. Select a starting value for p1 in the Acquisition params window.
- Determine the peak maximum for each pulse length iteration to determine the 90º pulse length.
- Determine the frequency of the signal, re-tune the coil, and change the frequency in the program. Repeat the 90º pulse length measurement.
- Change the amplifier power level until you obtain a pulse length of 1-2 ms.

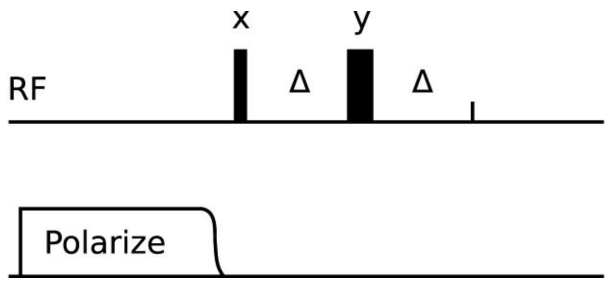

Figure 13. Spin-echo experiment (3). The narrow black bar represents a 90º RF pulse (with phase x), and the wide black bar a 180º pulse. Data acquisition takes place during the entire experiment (after polarization).

Acquiring an FID

- Change the parameters to acquire a single FID with optimal pulse length.
- Acquire an FID. You may use signal averaging ('nav' in the Iterations params) to achieve a higher signal-to-noise ratio.
- Experimentally optimize the orientation of the coil until you obtain maximum signal.
- During the acquisition, using the appropriate Hall probe, measure the polarization field.

Questions (1 and 2 should be done in the lab)

1. Calculate the resonance frequency from the field measurement.
2. Load the data in the Jupyter notebook (week1.ipynb). Work within the notebook to produce a publication-quality plot of the tuning response signal that includes all necessary labels, etc. Labels can be added in Python or using a separate program to open the image file.
3. Calculate the inductance of the coil.
4. Plot the peak maximum against the pulse length for two different amplifier power levels. Indicate the 90º pulse length in the plot.
5. Plot the echo and the Fourier transformed spectrum.
6. NMR is a precise method for measuring the magnitude of a magnetic field. Use the resonance frequency to calculate the exact magnitude of the earth's magnetic field at the location of the spectrometer. Is the precession frequency dependent on the orientation of the spectrometer? Why or why not?
7. You have used “oversampling” for acquiring the FID. Why is oversampling needed?
8. What is the voltage of the signal induced by the nuclear spins in the coil?
9. Calculate the equilibrium polarization of a proton spin system in the polarization field that you have measured, and in the earth’s field. What is the significance of the difference?
10. Calculate the sample magnetization after the sample has been polarized, and compare to the magnetic field strength $H = B / \mu_0$ of the earth. What makes it easy to detect the small magnetization of the water in the much larger magnetic field of the earth?

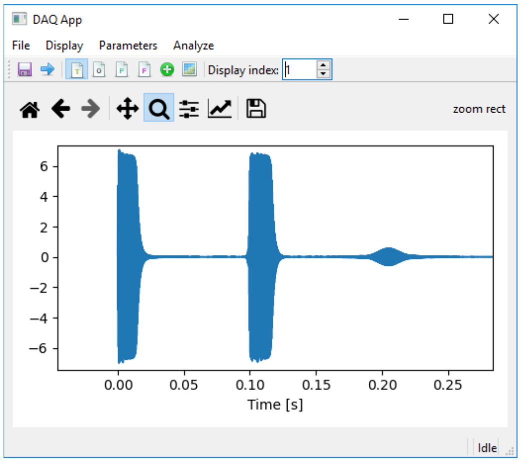

Figure 14. Time-domain data display from a spin-echo experiment. Visible are the 90º excitation pulse and ringdown near 0 s, the 180º refocusing pulse with ringdown near 0.1 s, and the signal from the spin echo near 0.2 s. The signal was processed with a digital filter to remove low-frequency interference.

Bonus Question

11. Define the term “Q-factor” and calculate its value from the tuning response signal (12). Why is the Q-factor an important parameter of the resonant circuit?

Week 2: Spin Relaxation

Two different types of spin-relaxation have been introduced above. In this lab, you will set up experiments to measure both of them. You will then use them to study the influence of paramagnetic substances and other sample properties on spin-relaxation.

Prelab Questions

1. For the T_1 experiment, what is the equation for the signal strength in function of the time during which a sample is pre-polarized ("Polarize" segment in Figure 13)?
2. For the T_2 experiment, what is the equation for the signal strength in function of the total echo time ($2n\Delta$ in Figure 16)?
3. Estimate τ_c for water, based on equation 18. Convince yourself that $\omega\tau_c << 1$ for the earth field NMR experiment. Simplify equation 20 for the case where $\omega\tau_c << 1$, and combine the simplified version with equation 18 to express R_1 in function of the viscosity.
4. Why is Gd-DTPA paramagnetic?

Measuring the spin-lattice relaxation time T_1

- Starting from the experiment that you used in the previous week, create a parameter set that can carry out a measurement of T_1 at the polarization field by incrementing the time the polarization field is turned on, but keeping the pulse length constant (see Figure 13). In order to do this, you need to increment 'ppre.poltime' in the Increment params window.

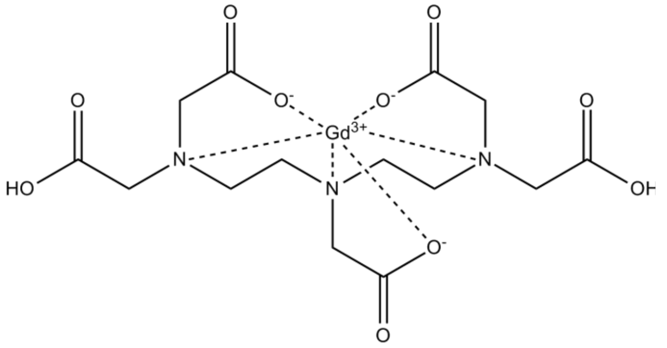

Figure 15. Gadolinium(III)-diethylenetriaminepentaacetic acid (Gd-DTPA)

- Use a Jupyter notebook to plot the function from prelab question 1, assuming a T_1 relaxation time of a few seconds. Refer to Appendix 1 for an example of how to plot a function using Python in Jupyter. Use the graph to determine suitable time points to be measured in order to yield a good fit. Also, increase the recycle time ('d' in Increment params) to 15 s to ensure that you are starting with a fully "relaxed" sample for every scan.
- Measure the relaxation of **water, and 10%, 20%, 30% and 50% glycerol in water**.
- Measure the relaxation time of a concentration series of **0.125 mM, 0.25 mM, 0.375 mM and 0.5 mM Gd-DTPA** (Figure 15) in water.

Spin-echoes and spin-spin relaxation time T_2

As opposed to the spin-lattice relaxation time, which measures the buildup or loss of polarization by reaching a thermodynamic equilibrium, the spin-spin relaxation is due to the de-phasing of a coherence. As discussed above, this process happens both due to macroscopic inhomogeneities, and due to fluctuations at the molecular level, giving rise to T_2* and to T_2. While T_2* can be measured directly from the decay of an FID, T_2 is only visible if the macroscopic inhomogeneities are first re-focused. This is done using a "train of echoes" (Figure 16). This scheme is termed the CPMG (Carr-Purcell-Meiboom-Gill) sequence (3), after the names of its inventors.

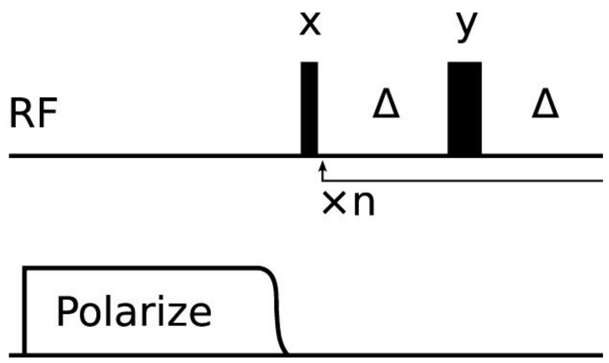

Figure 16. Pulse sequence for T_2 relaxation measurement at earth field.

- Again, starting from the previously saved experiment, generate a program that can be used for measuring T_2. Use a time Δ on the order of 50 ms.
- In Jupyter (see Appendix 1), plot the equation from prelab question 2, assuming a T_2 time constant on the same order as the T_1 time constant. Use the plot to decide on the number of echoes that should be acquired in order to obtain a good fit. The signal should have completely relaxed (disappeared) at the end of the echo train.
- Run the experiment and locate the spin echoes. They appear as rising and falling signals *between* the pulses. Their intensity decreases as a function of time. The echoes can be seen more clearly, if the FID is "blanked", such that the RF pulse and ringdown is removed. A digital filter can be applied to remove low-frequency interference from building electrical systems in the time domain data. Both the blanking and digital filter options are in the Analyze menu. They can also be accessed from within a Jupyter notebook.
- Once you have blanked the pulses, you may further generate an audio file from the time domain data, which can be played using a media player.
- Measure T_2 of the samples that you have previously used to measure T_1.

Questions (1 and 2 should be done in the lab)

1. Open the data in the Jupyter notebooks (week2a.ipynb and week2b.ipynb). Use the equation from the pre-lab questions to plot and fit this data. From the fit, determine the T_1 and T_2 relaxation time constants of all the samples.
2. Plot a "train of echoes" (Blanked FID)
3. Estimate T_2* for the water sample. What does T_2* predominantly depend on? Why is it of less interest than the actual T_2?
4. The spin-echo has historically been perplexing to scientists, as it apparently violates the second law of thermodynamics. Specifically, it appeared impossible that an ordered

state of the system (coherently precessing spins that give an NMR signal) can decay into a random state with no NMR signal, but then be "brought back". How can this be explained? (see the section on "The Spin-Echo Sequence" in (2)).

5. For the glycerol/water samples, compare your relaxation data with the equation that you have obtained in prelab question 3, and use this result to explain the trends observed based on relaxation theory.
6. An observant user may remark that T_1 was measured at the polarization field, while T_2 was measured at earth field. How could the pulse sequence for the T_1 measurement be changed so that T_1 is also measured at earth field?
7. For the samples with Gd-DTPA, plot the relaxation rate $R_1 = T_1^{-1}$ and $R_2 = T_2^{-1}$as a function of radical concentration. What function approximates this curve? – Fit your data and give the empirical equations describing the paramagnetic relaxation enhancement (PRE) of R_1 and R_2.
8. In medical MRI, variations of T_1 or T_2 yield contrast of the image. Specifically, T_1 contrast is achieved by using a polarization time short enough so that spins with a longer relaxation time do not fully polarize (e.g. $t_{polarize}$ = 500 ms). These spins then yield a lower signal in the MRI image. In addition to T_1 variations intrinsically present in tissue, a popular way of enhancing this contrast is through the use of Gd-DTPA as a contrast agent. (see for example (6)). Based on your relaxation data, estimate the amount of Gd-DTPA that should be used in a patient to achieve a contrast of 4:1 between a region accessible and a region not accessible to the contrast agent.

Bonus Experiment

If you have time, try to implement the change mentioned in question 6.

Week 3: Diffusion

Prelab Questions

1. Find the Stokes-Einstein equation
2. Combine the Stokes-Einstein with the equation found in prelab question 3 of week 2, to express T_1 in function of the diffusion coefficient D.

Diffusion measurements

Pulsed field gradients (PFGs) are controllable spatial variations of the magnetic field. By changing the precession frequency dependent on position, they enable distinguishing between different positions in space. Apart from magnetic resonance imaging (MRI), an easy experiment making use of PFGs is the measurement of diffusion.

The diffusion experiment (Figure 17) is a spin-echo experiment, to which two PFGs have been added (13). During the first PFG, spins in different places precess with different frequency (equation 22). After the PFG is turned off, the precession frequency is restored to the original value. However, from the precession during the PFG, different spins have acquired different phase. Therefore, the first gradient encodes the spatial position of each molecule into the phase of precession of its nuclear spins. Since the coherence is dephased, a signal is not observable at this point. After the time Δ, the second PFG following a 180º RF pulse has the inverse effect on the phase and refocuses the coherence. A signal is once again observable. However, if molecules change their position due to diffusion during Δ, the second gradient pulse will not completely refocus the coherence, and the observed signal is attenuated as a function of Δ and gradient strength.

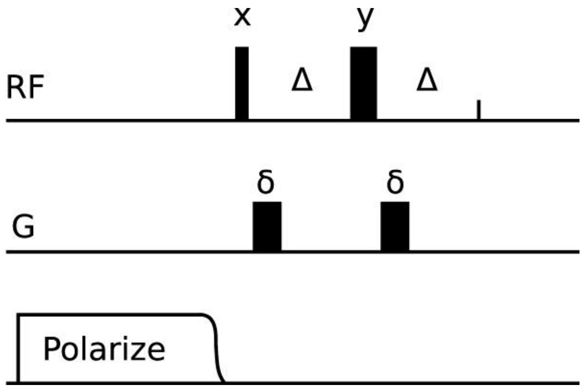

Figure 17. Pulse sequence for diffusion measurement.

$$M = M_0 e^{-\gamma^2 G^2 \delta^2 (\Delta - \delta/3) D}. \quad (24)$$

As opposed to most other methods for measuring diffusion, NMR is able to measure the true self-diffusion coefficient, in this case describing the diffusion of protons in water.

- Change the experiment parameters to generate the diffusion pulse sequence in Figure 17. Specifically, use the pulse program named diff. Add z-gradients with amplitude 1, and of incremented duration (0 – 19 ms in 1 ms steps).
- Measure the diffusion in **water and in 10% glycerol, 20% glycerol, 30% glycerol and 50% glycerol in water**. Use maximum setting for the z-gradient amplifier (knob on the upper amplifier). Use signal averaging as necessary.
- Measure diffusion in **toluene**.

Questions (1 and 2 should be done in the lab)

1. Calibrate the gradient strength G from your measurement of self-diffusion in water and the literature value for the diffusion coefficient of water. For fitting, you may use the Jupyter notebook (week3.ipynb).
2. Determine the diffusion coefficient of the samples you have measured, using the calibration from question 1.
3. Use the Stokes-Einstein relation (prelab question 1) to estimate the viscosity of toluene.
4. Compare the obtained viscosities with literature values. Discuss sources of errors, of both conceptual and experimental origin. See in particular (3, 14).
5. For the glycerol/water mixtures, plot T_1 against D, and verify that the relationship from prelab question 2 holds.
6. Would it be better to use a measurement of T_1 or diffusion in order to determine viscosity?
7. What are industrial and biomedical applications for the measurements of diffusion and relaxation that you carried out in this lab?

References

(1) Akins, P.; de Paula, J. *Physical Chemistry, 8th ed*; Freeman, 2006.
(2) *The Basics of NMR*. http://www.cis.rit.edu/htbooks/nmr (accessed 2023-8-31).
(3) Claridge, T.D.W. *High-resolution NMR Techniques in Organic Chemistry, 2nd ed.*; Elsevier, 2009.
(4) Levitt, M. *Spin Dynamics: Basics of Nuclear Magnetic Resonance, 2nd ed.*; John Wiley & Sons, 2008.
(5) Kowalewski, J.; Mäler, L. *Nuclear Spin Relaxation in Liquids*; Taylor & Francis, 2006.
(6) Wood, M.L.; Hardy, P.A. Proton relaxation enhancement. *J. Magn. Reson. Imaging.* **1993**, *3* (1) 149-156.
(7) *Lessons in electric circuits*; http://www.ibiblio.org/kuphaldt/electricCircuits/ (accessed 2023-8-31)
(8) Shoemaker, D.; Garland, C.; Nibler, J. Chapter XVII: Electronic Devices and Measurements. In *Experiments in Physical Chemistry, 8th ed.*; McGraw-Hill, 2008.
(9) Callaghan, P.; Eccles, C.; Seymour, An earth's field nuclear magnetic resonance apparatus suitable for pulsed gradient spin echo measurements of self-diffusion under Antarctic conditions. J. *Rev. Sci. Instr.* **1997**, *68* (11), 4263-4270.
(10) Callaghan, P.; Le Gros, M. Nuclear spins in the Earth's magnetic field. *Am. J. Phys.* **1982**, *50* (8), 709-713.
(11) Callaghan, P. *Principles of Nuclear Magnetic Resonance Microscopy*; Oxford University Press, 1994.
(12) Hoult, D. The NMR receiver: a description and analysis of design. *Prog. Nucl. Mag. Res. Sp.* **1978**, *12* (1), 41-77.
(13) Price, W.S. Pulsed-field gradient nuclear magnetic resonance as a tool for studying translational diffusion: Part 1. Basic theory. *Concepts Magnetic Res.* **1997**, *9* (5), 299-336.
(14) Chen, H.C.; Chen, S.H. Diffusion of crown ethers in alcohols. *J. Phys. Chem.* **1984**, *88*(21), 5118-5121.

Constants

Planck's constant:	$\hbar = 1.0546 \cdot 10^{-34} Js$
Boltzmann constant:	$k = 1.3807 \cdot 10^{-23} JK^{-1}$
Proton gyromagnetic ratio:	$\gamma_H = 2.6752 \cdot 10^{8} s^{-1} T^{-1}$

Appendix 1: Using Python and Jupyter

Background

This lab uses Python version 3 and Jupyter software for processing the data that was measured. Both are open source and completely free to install on your computer. Versions for Windows, Mac OS and Linux exist.

Python (https://www.python.org) is a general-purpose coding language, which is broadly used in science and engineering. There are numerous packages for python that contain functions for different tasks. Examples are numpy (functions for numerical and matrix calculations), scipy (functions for scientific computation), matplotlib (functions for plotting), and others. A package manager, either pip or Anaconda, can be used to most conveniently install these packages. Python is an interpreted language, which means that a Python program can be run without requiring a compilation step.

Jupyter (https://jupyter.org) is a software for the creation of interactive notebook style documents that contain integrated calculations, text comments, graphs and other elements. Code can be executed directly within the notebook. Jupyter works with several different coding languages; here, we use it in combination with Python. Jupyter comes in two flavors, the newer Jupyter Lab and the original Jupyter Notebook. The data analysis tasks in this lab work with either one. Jupyter can be installed locally on a computer, and once installed will run within the web browser.

Getting the Software

Jupyter Notebook, Python and the required packages are already installed in the **TAMU Open Access Labs** (https://oal.tamu.edu). They can be accessed by visiting a lab in person or by connecting to the Virtual OAL remotely. **If you plan to use the Open Access Lab, no further software installation is required.** You will need to transfer the data using a USB memory stick or through the internet.

If you will use your own computer, you need to install Python and Jupyter:

- If you do not yet have Python installed, download the latest version of Python 3 from https://www.python.org. Run the installer and follow the instructions. Allow all steps recommended by the installer (for example, the Windows installer recommends enabling long path names, which is required for the program to work).
- Open a terminal or command window (in Windows, this is accessed by typing cmd in the start menu). One after the other, type the following commands to install the required packages:

```
py -m pip install numpy
py -m pip install scipy
py -m pip install matplotlib
py -m pip install os
py -m pip install json
```

  - The py command runs python. Depending on your operating system and installation, the name for this command may instead be python3 or python. Replace as appropriate.

- If you have previously installed python using Anaconda, do not use the above commands. Rather, use the Anaconda package manager to install the corresponding packages. Mixing of pip packages with Anaconda may not work.

- If you do not yet have Jupyter installed, visit https://jupyter.org/install and follow the instructions (notice that they are for pip. For Anaconda based installations, the conda package manager should be used).
    - For a fresh installation, we recommend installing JupyterLab. If you have previously installed Jupyter Notebook, this will work as well.

Working with Python in Jupyter

- Test the installation by launching JupyterLab and creating a new notebook using the Python 3 kernel. Type the following and execute the commands:

```
import numpy as np
import matplotlib.pyplot as plt
t=np.linspace(0,10*np.pi,1000)
y=np.sin(t)
plt.plot(y)
```

  Execute each cell individually using Shift-Enter. These commands import the numpy and matplotlib.pyplot libraries, then graph a sin function for t=0…10pi, using 1000 points. When all commands are executed, you should see the graph in Figure A1.1 appear. In the above code, the functions designated with np. are from the numpy package. Details can be found in the documentation: https://numpy.org/doc/stable/user/index.html#user.

- Experiment with modifications of the graph. You can use the commands plt.xlabel and plt.ylabel to add labels to the axes.
- Review the matplotlib pyplot tutorial to learn about various display and formatting options for plots: https://matplotlib.org/stable/tutorials/introductory/pyplot.html#sphx-glr-tutorials-introductory-pyplot-py

- In the lab, four different Jupyter notebooks are provided to help analyze the data: Tuning and Pulse length determination (week 1); T1 measurement (week 2a); T2 measurement (week 2b); Diffusion measurement (week 3). The files require interactive adjustments to obtain the plots and analyzed data values, as indicated in each file.
- Using the Jupyter files requires the provided nmrbase library files, such that the same processing functions are available as in the data acquisition program. **The nmrbase folder needs to be placed as a subfolder within the folder containing the Jupyter notebook files.** This is regardless of whether you are using the python installation in the Open Access lab or on your own computer.

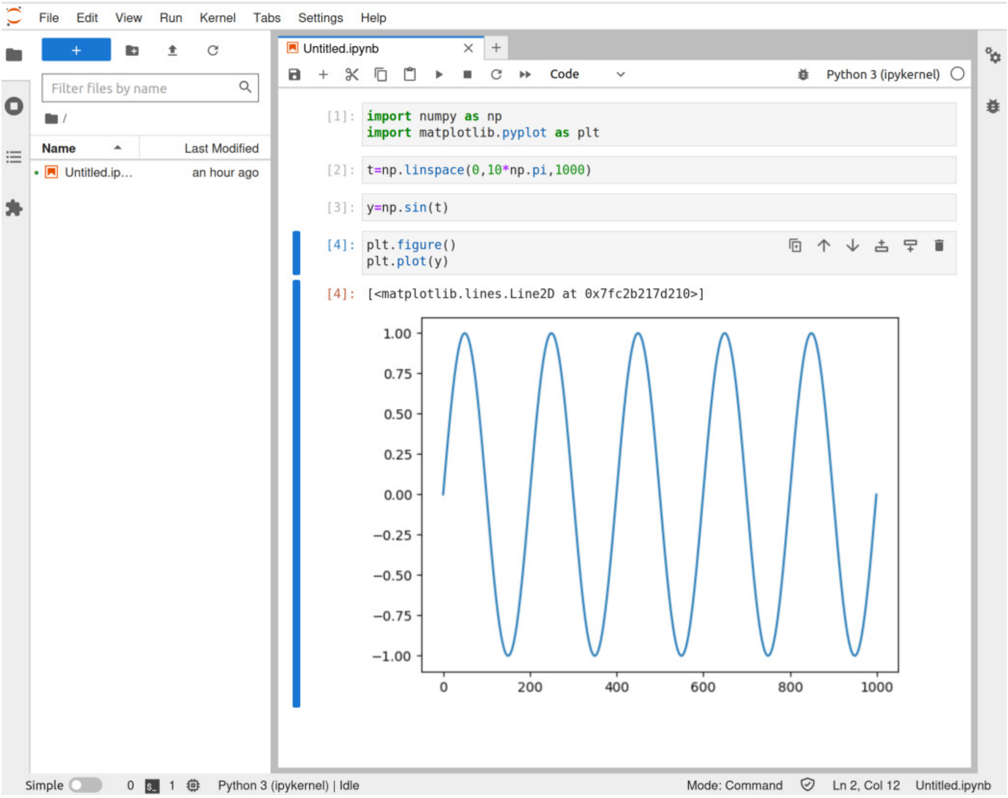

Figure A1.1. Example of using Jupyter Lab in Firefox. The notebook displays a graph of y=sin(t) for t from 0 to 10π, using 1000 data points.

Appendix 2: Data Acquisition Program

The NMR experiments are performed using the data acquisition program installed on the computer in the lab. The program is started by right-clicking on daqapp2.py, then selecting Open with/Python.
The data acquisition program is needed only in the lab. For the data analysis, libraries from this program are provided to use with the Jupyter notebooks (see above).
The typical workflow to use the program is to

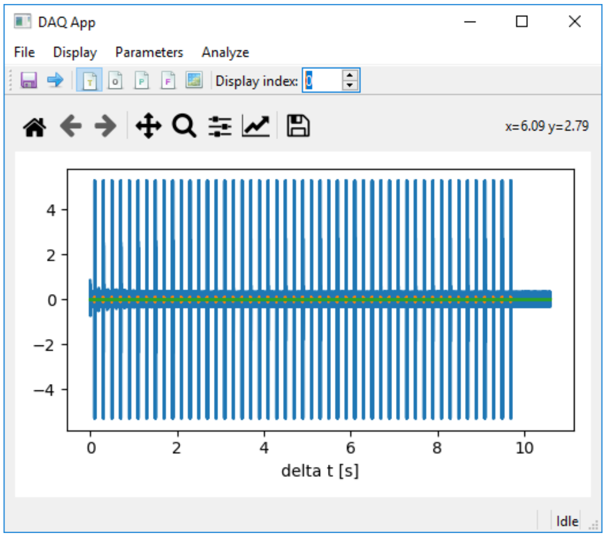

Figure A2.1. Main window of data acquisition program. The window shows the result of a signal acquisition with multiple echoes. The spin echoes appear in-between the large amplitude spikes from the RF pulses.

1. Load an existing data set that contains parameter definitions for the experiment.
2. Inspect the pulse program (Display/Display pulse program)
3. Adjust experimental parameters as needed (Parameters/Pre-acquisition parameters, Acquisition parameters, Increment parameters).
4. Run the experiment using File/Run (single scan) or File/Run experiment (multiple scans as defined by Iteration parameters).
5. If the Run option was used, save the data. This is not necessary for the Run experiment option, which automatically saves the data.
6. If the Run experiment option was used, re-load the data. This is necessary for experiments with multiple scans, since the program keeps only one scan in memory during the acquisition.
7. Adjust processing parameters (Parameters/Processing Parameters). Save the experiment with updated processing parameters if those should be kept for further analysis and plotting in Jupyter.
8. Perform any manual processing options (Analyze/Average Data, Analyze/Split Scan).
9. Inspect time-domain data (Display/Display time, Display/Display frequency, Display/Display integrals). In the toolbar, the Display Index can be used to flip through individual scans.

If for any reason the program terminates unexpectedly, restart the program and run another experiment to make sure all outputs are in the defined off-condition at the end.

Menus, Toolbars and Parameters

The menu and toolbar options are for accessing the configuration and experiment control functions.

- File menu
    - Export: Exports the displayed data in a format that can be read by a spreadsheet program. See processing parameters to change the traces that are displayed.
    - Export Audio: Export .waf audio file of the time domain data from the first trace.
    - Save: Saves the time-domain data and parameters. If the data has been modified, such as by the split function, the modified data is saved. Two files are created. The file with extension .json contains the parameters, while the file with extension .bin contains the data.
    - Load: Load saved time-domain data and parameters. Loading requires the corresponding .json and .bin files. Either one of the corresponding file names can be selected to load both.
    - Run: Runs a single scan of an experiment. Only the first scan is measured if increment parameters are defined. The data is not automatically saved.
    - Run experiment: Runs all scans of an experiment according to the increment parameters. The data is automatically saved to the file specified in the file dialog box that appears before the experiment runs.
    - Abort: Abort a multi-scan experiment after completion of the next scan. The data acquired up to this point remains saved.
    - Clear data: Clear the data in memory, while keeping the parameters.
- Display menu
    - Display time: Display the time domain data in the main window.
    - Display pre-program: Display the pre-polarization waveform from the pre-program in the main window.
    - Display pulse program: Display the generated pulse program in the main window. If increment parameters are specified, the pulse program that is displayed is for the first scan.
    - Display frequency: Display the Fourier transform, *i.e.*, the spectrum, in the main window.
    - Display integrals: Displays peak integral values that are integrated according to the processing parameters.
    - Display 2D spectrum: Display a transformed two-dimensional

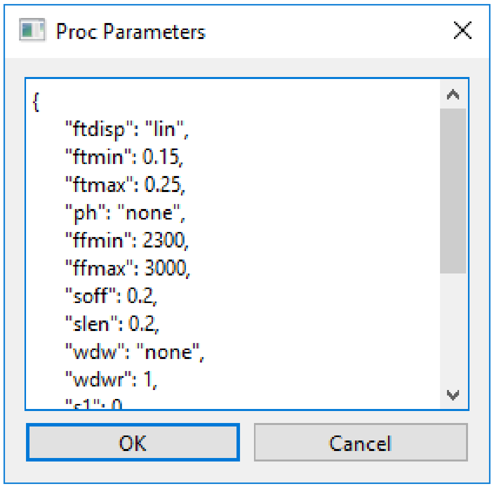

Figure A2.2. Processing parameters window. When changing parameters, the parentheses, commas and quotation marks need to be specified as shown.

spectrum in the main window (not used in this lab).

- Parameters menu
  The following lists describe the parameters that can be set in the different parameter windows. Not all parameters need to be changed in each experiment. The best practice is to load an existing data set and change only the parameters as needed. **A non-exhaustive set of parameters that need to be changed frequently during the course of the experiments is highlighted in bold.**
  - Pre-acquisition params: Display and edit the parameters for the pre-polarization wave form in the pre-program.
    `prog [name of pre-polarization program]`
    `aochann [hardware channel for pre-polarization waveform output]`
    `dochann [hardware channel for pre-polarization relay switching]`
    `polamp [amplitude for pre-polarization waveform /V]`
    `polsrate [sample rate for pre-polarization waveform /s^-1]`
    `poltime [time for pre-polarization /s]`
    `polramptime [time for pre-polarization rampdown]`
    `posttime [time to wait after pre-polarization and before pulse program /s]`
    `tpre [time to wait before start of pre-polarization /s]`
  - Acquisition params: Display and edit the parameters for the pulse program.
    `nsamp [number of samples]`
    `srate [sample rate /Hz]`
    `timeout [acquisition timeout /s]`
    `inchann [input channel of form /Dev1/ai0]`
    `inclk [sample clock of form /Dev1/ao/SampleClock; none for boards without internal synchronization]`
    `outchann [output channel of form /Dev1/ao0]`
    `pp [pulse program name, such as cpmg]`
    `aitrigg [input line to trigger analog input, of form /Dev1/PFI0]`
    `aotrigg [input line to trigger analog output, of form /Dev1/PFI1]`
    `triggdo [output line to send a trigger signal, of fomr /Dev1/port0/line0]`
    `nskip [acquired data points to skip]`
    `p1 [pulse length /s]`
    `amp [pulse amplitude /V]`
    `frq [pulse frequency /Hz]`
    `necho [number of echoes]`
    `tau [half echo time /s]`
  - Processing params: Display and edit parameters that pertain to the data display, Fourier transform, and other processing options.
    `ftdisp ['lin' or 'log' for linear or logarithmic FT display]`
    `ftmin [start time for ft /s]`
    `ftmax [stop time for ft; use 0 for full range /s]`
    `ph [phase correction for FT /rad. "none" = magnitude spectrum]`
    `ffmin [start of ft display /Hz]`
    `ffmax [end of ft display /Hz]`
    `soff [offset for split /s]`

slen [length for split /s]
wdw [window function / "none" or "exp"]
wdwr [decay constant for exponential window / s^-1]
s1 [lower signal limit /Hz]
s2 [higher signal limit /Hz]
n1 [lower noise limit /Hz]
n2 [higher noise limit /Hz]
sind [index for snr display]
fc [process percentage of range, centered]
autosplit ["true" to use pulse program defined function to split]
disp [array of traces to display]
blankb [border region for pulse blanking /s]
intmin [lower frequency of integral region /Hz]
intmax [higher frequency of integral region /Hz]
digfmin [lower cutoff frequency for digital filter /Hz]
digfmax [higher cutoff frequency for digital filter /Hz]

- Increment params: Display and edit the parameters that should be incremented in multi-scan experiments.
nav [number of averages (not saved separately) /integer]
n [number of increments for loops /array of integer]
incp [parameter to increment, by default from the acquisition parameters window. To specify a parameter from the pre-acquisition parameters, use the ppre. prefix, e.g. ppre.poltime /array of string]
inc [increment value /array of number]
d [time added between increments /s]
- Status params: These parameters are saved once the experiment is run. They cannot be changed manually.
ninchann [number of input channels]
navdone [number of averages performed]
skip [number of data points omitted]
nsampx [actual number of samples]
sratex [actual sample rate /s^-1],
ioff [actual offset index for split function]
ilen [actual data length for split function]

- Analyze menu
  - Average data: Replaces the data in memory with the average of all scans.
  - Blank pulses: Replaces the time domain data with a blanked version, where the non-zero regions from the pulse program, plus border region according to processing parameters, are set to zero.
  - Digital filter: Replaces the time domain data with a version that is digitally filtered with a 4-th order Butterworth filter, and frequency cutoff values according to the processing parameters.
  - Split scan: Splits a single scan into multiple pieces, according to the corresponding processing parameters.
- Toolbar
  - Icons: The function of the icons is the same as in the menu options.
  - Display index: Select the index of the scan that should be displayed. The index 0 shows a concatenated display of all scans. Because the concatenated display does not represent actual time or frequency, the horizontal axis is labeled as delta t or delta f in this case. The actual time

or frequency is seen in an individual indexed display (index 1 or larger). If there is only one scan, the data for index 0 and index 1 are the same.

- The integral display shows integrals for all scans in a single graph, hence the display index does not have an influence on the integral display.

Appendix 3: Questions for Final Exam

1. Draw a diagram of the experimental setup.

2. Draw and explain the pulse sequence experiment you used for measurement of the spin-spin relaxation time T_2.

3. Draw the experiment that you used for measurement of the spin-lattice relaxation time T_1.

4. Draw and explain the pulse sequence experiment you used for measurement of diffusion.

5. Draw and explain the pulse sequence experiment you used to determine the 90-degree pulse.

6. What is the difference between T_1 and T_2 relaxation?

7. Explain the difference between a coherent state of a spin system and a polarized state of a spin system.

8. Explain how you tuned the rf coil to the frequency needed for NMR measurement.

9. Explain the effect of a free radical on spin relaxation.

week1

August 23, 2023

Notebook file for Week 1

Import libraries. The nmrbase folder needs to be located within the folder containing this Jupyter notebook

[ ]:
```
import numpy as np
import matplotlib.pyplot as plt
import nmrbase.expbase as expbase
import nmrbase.expdta as expdta
```

Tuning response experiment

[ ]:
```
filename = r"../DIRECTORY/FILENAME"   #Defines the path to the data file. Here␣
↪the path is relative to the current folder.
```

[ ]:
```
a = expbase.expbase()                         # create an experiment base object (a)
a.load(filename)                              # load data into a
f1 = plt.figure()
ax1 = f1.subplots()              # create axes for a figure

a.plottm(ax1,1)                               # plot the time domain data of the␣
↪first scan from the data set
```

[ ]:
```
## ADDITIONAL TASKS:
## include statements such as ax1.set_ylabel() to label axes
## use statements to change appearance such as font size, etc.
## Adjust horizontal axis limits to display only the ring-down signal with␣
↪statements such as ax1.set_xlim([0,1]). You will need to select appropriate␣
↪values.

f1    # show the figure again with modifications
```

[ ]:
```
## TASKS:
## change parameter "ftmin" and "ftmax" to select the time interval␣
↪corresponding to the ringdown for the Fourier transform
## change parameter "ffmin" and "ffmax" to select the frequency range of the␣
↪tuning response signal
## include statements to label axes
## use statements to change appearance such as font size, etc.
```

```
## Adjust horizontal axis limits to display the spectrum of the ring-down signal

f2=plt.figure()
ax2=f2.subplots()

a.pproc['ftmin']=VALUE                          # time interval for Fourier␣
 ↪transform (0 = all data)
a.pproc['ftmax']=VALUE
a.pproc['ffmin']=VALUE                          # frequency interval for spectrum␣
 ↪display (0 = all data)
a.pproc['ffmax']=VALUE
a.proc()                                 # calculate Fourier transform of the data␣
 ↪in a

a.plotfrq(ax2,1)                         # plot the frequency domain data of the␣
 ↪first scan from the data set
```

[ ]:
```
i=np.argmax(a.frq[0].dta)      # find data point index of largest peak in spectrum
f=a.frq[0].ind_to_x(i)         # find frequency corresponding to largest peak in␣
 ↪spectrum
print("Tuning frequency: {:.2f} Hz".format(f))      # this statement prints the␣
 ↪identified frequency in a nice format
```

[ ]:
```
# plot figure again with line indicating maximum peak position. Notice this␣
 ↪works only if previous parameters have been correctly selected.
ax2.axvline(x = f, color = 'r', label = 'Larmor Frequency')

f2   # show the figure again with the line included
```

Pulse length calibration. NOTE: To analyze and report multiple datasets, simply copy the necessary blocks of code and change the name of the veriables.

[ ]:
```
filename2 = r"../DIRECTORY/FILENAME"         # path of data file
```

[ ]:
```
a2 = expbase.expbase()
a2.load(filename2)                      # load data

f3=plt.figure()
ax3=f3.subplots()
a2.plottm(ax3,5)
```

[ ]:
```
## TASKS:
## fine tune with ax3.set_xlim and ax3.set_ylim parameters to zoom in on the echo

ax3.set_ylim([VALUE,VALUE])
ax3.set_xlim([VALUE,VALUE])
```

```
f3   # display adapted figrue
```

[ ]:
```
a2.pproc['digfmin']=1500     # set appropriate digital filter parameters
a2.pproc['digfmax']=3500
a2.digfilt()                 # perform digital filter operation
```

[ ]:
```
f4=plt.figure()
ax4=f4.subplots()
a2.plottm(ax4,5)   # plot the 5th scan (the 1st scan doesn't have signal because␣
↪the pulse is too short)
```

[ ]:
```
## TASKS:
## fine tune with ax4.set_xlim and ax4.set_ylim parameters to zoom in on the echo

ax4.set_ylim([VALUE,VALUE])
ax4.set_xlim([VALUE,VALUE])

f4   # display adapted figrue
```

[ ]:
```
f5=plt.figure()
ax5 = f5.subplots()

a2.pproc['ftmin']=VALUE                        # time interval for Fourier␣
↪transform (0 = all data)
a2.pproc['ftmax']=VALUE
a2.pproc['ffmin']=VALUE                        # frequency interval for spectrum␣
↪display (0 = all data)
a2.pproc['ffmax']=VALUE
a2.proc()                               # calculate Fourier transform of the data␣
↪in a
## this will use the digitally filtered data from before. Instead, the original␣
↪data can be processed by loading it again.

a2.plotfrq(ax5,5)                       # plot the frequency domain data of the␣
↪first scan from the data set

## TASKS:
## change parameter "ftmin" and "ftmax" to select the time interval␣
↪corresponding to the ringdown for the Fourier transform
## change parameter "ffmin" and "ffmax" to select the frequency range of the␣
↪tuning response signal
## include statements to label axes
## use statements to change appearance such as font size, etc.
## Adjust horizontal axis limits to display the spectrum of the ring-down signal
```

[ ]:

```
# This section is to integrate the peaks in the NMR spectra to determine pulse␣
↪length

## TASKS:
## change the intmin and intmax parameters to select the correct frequency range␣
↪for integration
## use set_xlabel and set_ylabel to set the labels to get a publication quality␣
↪figure
## adjust appearance of figure as needed
## change and report the "x" parameter to indicate the pulse length for 90°␣
↪pulse with a vertical line

f5=plt.figure()
ax5=f5.subplots()

a2.pproc['intmin']=VALUE      # set correct frequency range for integration
a2.pproc['intmax']=VALUE

a2.integrate()               # perform integration

#find the starting pulselength and increment, then set the correct x-axis
dx=a2.pinc["inc"][0]
x0=a2.p["p1"]
print('x0 =',x0,'s , dx =',dx,'s')          # x0 is the starting pulse length,␣
↪and dx is the increment
a2.idt.x0=x0
a2.idt.dx=dx

a2.idt.plot(ax5,disp=[0])                   # disp=0 plots only the NMR signal␣
↪in trace 0
ax5.axvline(x = VALUE, color = 'r', label = '90° pulse length') # display␣
↪vertical line at 90 degree pulse length
```

week2a

August 23, 2023

Notebook file for Week 2, T1 Relaxation experiment

Import libraries. The nmrbase folder needs to be located within the folder containing this Jupyter notebook

[ ]:
```
import numpy as np
import scipy
import matplotlib.pyplot as plt
import nmrbase.expbase as expbase
import nmrbase.expdta as expdta
```

T1 Relaxation NOTE: To analyze and report multiple datasets, simply copy the necessary blocks of code.

[ ]:
```
filename = r"../DIRECTORY/FILENAME"   #Defines the path to the data file. Here␣
↪the path is relative to the current folder.
```

[ ]:
```
a = expbase.expbase()
a.load(filename)                          # load data

f1=plt.figure()
ax1=f1.subplots()
a.plottm(ax1,5)
```

[ ]:
```
## TASKS:
## fine tune with set_xlim and set_ylim parameters to zoom in on the echo

ax1.set_ylim([VALUE,VALUE])
ax1.set_xlim([VALUE,VALUE])

f1
```

[ ]:
```
a.pproc['digfmin']=2000     # set appropriate digital filter parameters
a.pproc['digfmax']=3500
a.digfilt()                 # perform digital filter operation
```

[ ]:
```
## TASKS:
## fine tune with set_xlim and set_ylim parameters to zoom in on the echo
```

```
f2=plt.figure()
ax2=f2.subplots()

a.plottm(ax2,5)    # plot the 5th scan (the 1st scan doesn't have signal because␣
↪the polarization is too short)
ax2.set_ylim([VALUE,VALUE])
ax2.set_xlim([VALUE,VALUE])
```

[ ]:
```
## TASKS:
## change parameter "ftmin" and "ftmax" to select the time interval␣
↪corresponding to the echoes for the Fourier transform
## change parameter "ffmin" and "ffmax" to select the frequency range of the NMR␣
↪signal
## include statements to label axes
## use statements to change appearance such as font size, etc.

f3=plt.figure()
ax3 = f3.subplots()

a.pproc['ftmin']=VALUE                   # time interval for Fourier transform␣
↪(0 = all data)
a.pproc['ftmax']=VALUE
a.pproc['ffmin']=VALUE                   # frequency interval for spectrum␣
↪display (0 = all data)
a.pproc['ffmax']=VALUE
a.pproc['dispper']=0.2               # leave 20% space between each acquired␣
↪spectrum
a.proc()                             # calculate Fourier transform of the data␣
↪in a
## this will use the digitally filtered data from before. Instead, the original␣
↪data can be processed by loading it again.

a.plotfrq(ax3,0)                     # plot the frequency domain data of the␣
↪first scan from the data set
```

[ ]:
```
# Polarization-duration-resolved integration of peaks on NMR spectra

## TASKS:
## change the intmin and intmax parameters to select the correct frequency range␣
↪for integration
## use set_xlabel and set_ylabel to set the labels to get a publication quality␣
↪figure

f4=plt.figure()
ax4=f4.subplots()
```

```
a.pproc['intmin']=VALUE      # set correct frequency range for integration
a.pproc['intmax']=VALUE

a.integrate()                # perform integration

#find the starting polarization duration and increment, then set the correct␣
↪x-axis
dx=a.pinc["inc"][0]
x0=a.ppre["poltime"]
print('x0 =',x0,'s , dx =',dx,'s')           # x0 is the starting polarization␣
↪duration, and dx is the increment
a.idt.x0=x0
a.idt.dx=dx

a.idt.plot(ax4,disp=[0])                     # disp=0 plots only the NMR signal in␣
↪trace 0

ax4.set_xlabel("seconds [s]")
ax4.set_ylabel("Y LABEL")
```

Fitting of the integral to time axis. NOTE: For some results to fit, optimize the initial conditions

[ ]:
```
# Fitting of the integral

## TASKS:
## change the initial fitting parameters to obtain proper fitting
## report the fitted relaxation constants with proper significant figures
## use set_xlabel and set_ylabel to set the labels to get a publication quality␣
↪figure
## adjust appearance of figure as needed

ax5 = plt.figure().subplots()
a.idt.plot(ax5,disp=[0])                     # to be overlayed by fitted curve,␣
↪disp=0 plots only the NMR signal in trace 0

def fun(t,a,b,c):
    return FORMULA                           # INPUT the formula used for fitting.
↪ Use "np.exp()" for exponential, and t for time-axis.

y = a.idt.dta[0]    # Integral data points to fit
x = np.linspace(a.idt.x0,a.idt.x0+a.idt.dx*(a.pinc['n'][0]-1),a.pinc['n'][0])  ␣
↪# Time axis

a.p1,a.p2=scipy.optimize.
↪curve_fit(fun,x,y,p0=[A_VALUE,B_VALUE,C_VALUE],maxfev=5000)            #␣
↪Select the initial conditions
```

```
print('T\u2081 = ',round(1/a.p1[1],SF1),'±',round(np.linalg.eig(a.p2)[0][1]**0.
↪5,SF2),'s')

# REPLACE "SF1" and "SF2" with positive integrers to report the fitting results␣
↪with correct significant figures

x=np.linspace(x[0],x[-1],1000)                          # use 1000 points to␣
↪generate a smooth curve
a.fit_points = a.p1[0]*np.exp(-a.p1[1]*x)+a.p1[2]
pl,=ax5.plot(x,a.fit_points,'r-')

ax5.set_xlabel("seconds [s]")                        # SET the labels to get a␣
↪publication quality figure
ax5.set_ylabel("Y LABEL")
pl.figure.set_tight_layout('pad')
pl.figure.canvas.draw()
```

week2b

August 23, 2023

Notebook file for Week 2, T2 Relaxation experiment

Import libraries. The nmrbase folder needs to be located within the folder containing this Jupyter notebook

[ ]:
```
import numpy as np
import scipy
import matplotlib.pyplot as plt
import nmrbase.expbase as expbase
import nmrbase.expdta as expdta
```

T2 Relaxation NOTE: To analyze and report multiple datasets, simply copy the necessary blocks of code.

[ ]:
```
filename = r"../DIRECTORY/FILENAME"   #Defines the path to the data file. Here␣
↪the path is relative to the current folder.
```

[ ]:
```
a = expbase.expbase()
a.load(filename)                        # load data

f1=plt.figure()
ax1=f1.subplots()
a.split()                               # splitting raw T2 data for later␣
↪processes
a.plottm(ax1,0)
```

[ ]:
```
## TASKS:
## fine tune with set_xlim and set_ylim parameters to zoom in on the echo

ax1.set_ylim([VALUE,VALUE])
ax1.set_xlim([VALUE,VALUE])

f1
```

[ ]:
```
a.pproc['digfmin']=1500      # set appropriate digital filter parameters
a.pproc['digfmax']=3500
a.digfilt()                  # perform digital filter operation
```

[ ]:
```
## TASKS:
## fine tune with set_xlim and set_ylim parameters to zoom in on the echo

f2=plt.figure()
ax2=f2.subplots()

a.plottm(ax2,0)   # plot the entire scan

ax2.set_ylim([VALUE,VALUE])
ax2.set_xlim([VALUE,VALUE]) #zoom in on the 1st echo, which is the strongest one
```

[ ]:
```
## TASKS:
## change parameter "ftmin" and "ftmax" to select the time interval␣
↪corresponding to the echoes for the Fourier transform
## change parameter "ffmin" and "ffmax" to select the frequency range of the NMR␣
↪signal
## include statements to label axes
## use statements to change appearance such as font size, etc.

f3=plt.figure()
ax3 = f3.subplots()

a.pproc['ftmin']=VALUE                   # time interval for Fourier transform␣
↪(0 = all data)
a.pproc['ftmax']=VALUE
a.pproc['ffmin']=VALUE                   # frequency interval for spectrum␣
↪display (0 = all data)
a.pproc['ffmax']=VALUE
a.pproc['dispper']=0.5                 # leave 50% space between each acquired␣
↪spectrum
a.proc()                               # calculate Fourier transform of the data␣
↪in a
## this will use the digitally filtered data from before. Instead, the original␣
↪data can be processed by loading it again.

a.plotfrq(ax3,0)                       # plot the frequency domain data of the␣
↪first scan from the data set
```

[ ]:
```
# Time-resolved integration of peaks on NMR spectra

## TASKS:
## change the intmin and intmax parameters to select the correct frequency range␣
↪for integration
## use set_xlabel and set_ylabel to set the labels to get a publication quality␣
↪figure
```

```
f4=plt.figure()
ax4=f4.subplots()

a.pproc['intmin']=VALUE      # SET correct frequency range for integration
a.pproc['intmax']=VALUE

a.integrate()               # perform integration

#find the waiting time tau, then set the correct x-axis
dx=a.pproc["soff"]
x0=a.pproc["slen"]
print('x0 =',x0,'s , dx =',dx,'s')          # x0 is the time of 1st echoes, and␣
↪dx is the interval between echoes
a.idt.x0=x0
a.idt.dx=dx

a.idt.plot(ax4,disp=[0])                   # disp=0 plots only the NMR signal in␣
↪trace 0

ax4.set_xlabel("seconds [s]")
ax4.set_ylabel("Y LABEL")
```

Fitting of the integral to time axis. NOTE: For some results to fit, optimize the initial conditions

[ ]:
```
# Fitting of the integral

## TASKS:
## change the initial fitting parameters to obtain proper fitting
## report the fitted relaxation constants with proper significant figures
## use set_xlabel and set_ylabel to set the labels to get a publication quality␣
↪figure
## adjust appearance of figure as needed

ax5 = plt.figure().subplots()
a.idt.plot(ax5,disp=[0])                   # to be overlayed by fitted curve,␣
↪disp=0 plots only the NMR signal in trace 0

def fun(t,a,b,c):
    return FORMULA                         # INPUT the formula used for fitting.
↪ Use "np.exp()" for exponential, and t for time-axis.

y = a.idt.dta[0]    # Integral data points to fit
x = np.linspace(a.idt.x0,a.idt.x0+a.idt.dx*round(len(y)-1),len(y))   # Time axis

a.p1,a.p2=scipy.optimize.
↪curve_fit(fun,x,y,p0=[A_VALUE,B_VALUE,C_VALUE],maxfev=5000)           #␣
↪Select the initial conditions
```

```
print('T\u2082 = ',round(1/a.p1[1],SF1),'±',round(np.linalg.eig(a.p2)[0][1]**0.
↪5,SF2),'s')              # REPLACE "SF1" and "SF2" with positive integrers to␣
↪report the fitting results with correct significant figures

x=np.linspace(x[0],x[-1],1000)                                 # use 1000 points to␣
↪generate a smooth curve
a.fit_points = a.p1[0]*np.exp(-a.p1[1]*x)+a.p1[2]
pl,=ax5.plot(x,a.fit_points,'r-')

ax5.set_xlabel("seconds [s]")                                 # SET the labels to get a␣
↪publication quality figure
ax5.set_ylabel("Y LABEL")
pl.figure.set_tight_layout('pad')
pl.figure.canvas.draw()
```

week3

August 23, 2023

Notebook file for Week 3, Diffusion experiment

Import libraries. The nmrbase folder needs to be located within the folder containing this Jupyter notebook

[ ]:
```
import numpy as np
import scipy
import matplotlib.pyplot as plt
import nmrbase.expbase as expbase
import nmrbase.expdta as expdta
```

Diffusion NOTE: To analyze and report multiple datasets, simply copy the necessary blocks of code.

[ ]:
```
filename = r"../DIRECTORY/FILENAME"    #Defines the path to the data file. Here␣
↪the path is relative to the current folder.
```

[ ]:
```
a = expbase.expbase()
a.load(filename)                           # load data

f1=plt.figure()
ax1=f1.subplots()
a.plottm(ax1,1)
```

[ ]:
```
## TASKS:
## fine tune with set_xlim and set_ylim parameters to zoom in on the echo

ax1.set_ylim([VALUE,VALUE])
ax1.set_xlim([VALUE,VALUE])

f1
```

[ ]:
```
a.pproc['digfmin']=1500      # set appropriate digital filter parameters
a.pproc['digfmax']=3500
a.digfilt()                  # perform digital filter operation
```

[ ]:
```
## TASKS:
## fine tune with set_xlim and set_ylim parameters to zoom in on the echo

f2=plt.figure()
```

```
ax2=f2.subplots()

a.plottm(ax2,1)   # plot the 1st scan
ax2.set_ylim([VALUE,VALUE])
ax2.set_xlim([VALUE,VALUE])
```

```
[ ]: ## TASKS:
## change parameter "ftmin" and "ftmax" to select the time interval␣
 ↪corresponding to the echoes for the Fourier transform
## change parameter "ffmin" and "ffmax" to select the frequency range of the NMR␣
 ↪signal
## include statements to label axes
## use statements to change appearance such as font size, etc.

f3=plt.figure()
ax3 = f3.subplots()

a.pproc['ftmin']=VALUE                   # time interval for Fourier transform␣
 ↪(0 = all data)
a.pproc['ftmax']=VALUE
a.pproc['ffmin']=VALUE                   # frequency interval for spectrum␣
 ↪display (0 = all data)
a.pproc['ffmax']=VALU
a.pproc['dispper']=0.2                 # leave 20% space between each acquired␣
 ↪spectrum
a.proc()                               # calculate Fourier transform of the data␣
 ↪in a
## this will use the digitally filtered data from before. Instead, the original␣
 ↪data can be processed by loading it again.

a.plotfrq(ax3,0)                       # plot the frequency domain data of the␣
 ↪first scan from the data set
```

```
[ ]: # Gradient-duration-resolved integration of peaks on NMR spectra

## TASKS:
## change the intmin and intmax parameters to select the correct frequency range␣
 ↪for integration
## use set_xlabel and set_ylabel to set the labels to get a publication quality␣
 ↪figure

f4=plt.figure()
ax4=f4.subplots()

a.pproc['intmin']=VALUE      # set correct frequency range for integration
a.pproc['intmax']=VALUE
```

```
a.integrate()                # perform integration

#find the starting gradient duration and increment, then set the correct x-axis
dx=a.pinc["inc"][0]
x0=a.p["p2"]
print('x0 =',x0,'s , dx =',dx,'s')          # x0 is the starting gradient␣
 ↪duration, and dx is the increment
a.idt.x0=x0
a.idt.dx=dx

a.idt.plot(ax4,disp=[0])                   # disp=0 plots only the NMR signal in␣
 ↪trace 0

ax4.set_xlabel("seconds [s]")
ax4.set_ylabel("Y LABEL")
```

Fitting of the integral to time axis. NOTE: For some results to fit, optimize the initial conditions

```
[ ]: # Fitting of the integral

## TASKS:
## change the initial fitting parameters to obtain proper fitting
## report the fitted gradient strength/diffusion coefficient with proper␣
 ↪significant figures
## use set_xlabel and set_ylabel to set the labels to get a publication quality␣
 ↪figure
## adjust appearance of figure as needed

ax5 = plt.figure().subplots()
a.idt.plot(ax5,disp=[0])                   # to be overlayed by fitted curve,␣
 ↪disp=0 plots only the NMR signal in trace 0
big_delta=a.p.get('tau',0.1)                # time interval between gradients

def fun(t,a,b):
    return FORMULA                          # INPUT the formula used for fitting.
 ↪ Use "np.exp()" for exponential, and t for small-delta (gradient duration).

y = a.idt.dta[0]
x = np.linspace(a.idt.x0,a.idt.x0+a.idt.dx*(a.pinc['n'][0]-1),a.pinc['n'][0])

a.p1,a.p2=scipy.optimize.curve_fit(fun,x,y,p0=[A_VALUE,B_VALUE],maxfev=5000)   ␣
 ↪            # SELECT the initial conditions, especially the exponential index

print(round(a.p1[1],SF1),'±',round(np.linalg.eig(a.p2)[0][1]**0.5,SF2))          ␣
 ↪           # REPLACE "SF1" and "SF2" with positive integrers to report the␣
 ↪fitting results with correct significant figures
```

```
x=np.linspace(x[0],x[-1],1000)                          # use 1000 points to␣
 ↪generate a smooth curve
a.fit_points = a.p1[0]*np.exp(-a.p1[1]*x**2*(big_delta-x/3))
pl,=ax5.plot(x,a.fit_points,'r-')

ax5.set_xlabel("seconds [s]")                         # SET the labels to get a␣
 ↪publication quality figure
ax5.set_ylabel("Y LABEL")
pl.figure.set_tight_layout('pad')
pl.figure.canvas.draw()
```
